# Tissue-specific moderate DNA methylation alterations featuring hypomethylation in microRNA regulatory regions associated with advanced carotid atherosclerosis

**DOI:** 10.3389/fcell.2026.1948449

**Published:** 2026-10-19

**Authors:** Iuliia A. Koroleva, Alexey A. Zarubin, Anton V. Markov, Alexey A. Sleptcov, Mikhail S. Kuznetsov, Boris N. Kozlov, Elvira F. Muslimova, Sergey A. Afanasiev, Nadezhda P. Babushkina, Elena Yu. Bragina, Irina A. Goncharova, Maria V. Golubenko, Aksana N. Kucher, Maria S. Nazarenko

**Affiliations:** 1 Research Institute of Medical Genetics, Tomsk National Research Medical Center, Tomsk, Russia; 2 Cardiology Research Institute, Tomsk National Research Medical Center, Tomsk, Russia

**Keywords:** Bisulfite amplicon sequencing, BSAS, carotid atherosclerosis, methylation, microrna genes, miRNA-seq

## Abstract

**Introduction:**

DNA methylation is an important epigenetic mechanism involved in the regulation of gene expression, but its contribution to the regulation of microRNA genes in atherosclerosis remains poorly understood.

**Methods:**

We profiled DNA methylation of regulatory elements and gene bodies of five microRNA genes (*MIR10B, MIR21, MIR100, MIR127, and MIR143*) in vascular tissues and paired peripheral blood leukocytes from 92 patients with advanced carotid atherosclerosis along with peripheral blood leukocytes of 32 subclinical atherosclerosis controls, using targeted bisulfite sequencing.

**Results:**

We identified distinct tissue-specific DNA methylation patterns for all studied regions of microRNA genes. The regulatory regions of *MIR10B, MIR127*, and *MIR100* were moderately hypomethylated in carotid atherosclerotic plaques compared with intact vascular tissues. We further integrated our findings with lab-internal and publicly available epigenome-wide methylation datasets and evaluated the contribution of vascular and blood cell composition using computational deconvolution. After adjustment for estimated vascular cell proportions, methylation at CpG sites within the *MIR100* and *MIR127* regions remained associated with atherosclerosis. Increased DNA methylation at a single CpG site (chr11:122025143, GRCh37/hg19) located in the *MIR100* E-box region was associated with metabolic syndrome, while DNA methylation levels of *MIR10B, MIR21*, and *MIR127* in atherosclerotic plaques were linked with indicators of histological instability and a history of acute cerebrovascular events. In peripheral blood cells, we observed moderate hypomethylation, predominantly at individual CpG sites within the regulatory regions of *MIR10B, MIR21*, and the *MIR100* E-box region in patients compared with controls. However, MIR10B methylation showed weak associations with estimated blood cell composition. In blood, *MIR10B* and *MIR143* methylation correlated with lipid metabolism and carotid stenosis, while the *MIR21* CpG island showed strong correspondence between blood and plaque methylation. Using miRNA-seq to analyze microRNA expression in vascular samples, we identified miR-143–3p, miR-21–5p, miR-100–5p, miR-145–5p, miR-145–3p, and miR-10b-5p as the most highly expressed microRNAs. Methylation of CpG sites within *MIR10B* and *MIR143HG* (*CARMN*) promoters was inversely associated with the expression of the corresponding microRNAs.

**Discussion:**

Advanced carotid atherosclerosis is characterized by specific moderate tissue-associated DNA methylation patterns of microRNA genes, predominantly involving hypomethylation of regulatory regions. These epigenetic shifts, driven both by changes in vascular cell composition and the functional state of the vessel wall, enable highly coordinated regulation of microRNA expression profiles. These findings highlight promising candidate loci and biomarkers for predicting the complicated course of atherosclerosis, which warrants further validation in larger independent cohorts.

## 1 Introduction

Atherosclerosis (AS) is a complex lesion of arteries characterized by chronic inflammation, lipid accumulation, and vascular wall remodeling. In advanced stages, rupture or erosion of atherosclerotic plaques triggers platelet adhesion, thrombus formation, arterial occlusion and acute ischemic events, including coronary artery disease, myocardial infarction, and ischemic stroke, which remain the leading cause of mortality worldwide (Volný et al., 2015; Li et al., 2023; Euler and Parahuleva, 2025).

The pathogenesis of AS is regulated by multiple classes of RNA molecules, including microRNAs (miRNAs), which are small single-stranded non-coding RNAs, typically 16–27 nucleotides in length, that regulate gene expression at the post-transcriptional level (Fang et al., 2013; Vartak et al., 2022; Wang et al., 2025). Approximately 2,500 miRNAs have been identified in the human genome, of which nearly 150 microRNAs play a critical role in the cardiovascular system (Cordeiro. et al., 2025).

MicroRNAs dichotomously regulate a wide range of biological processes in AS including initiation (endothelial senescence and inflammation), progression (SMC proliferation and migration, increased necrosis of lipid core, angiogenesis), and endpoint (unstable plaque rupture) (Wang et al., 2025). According to their effects on atherosclerosis progression, microRNAs can be divided into proatherogenic, antiatherogenic or ambigual groups (Volný et al., 2015; Euler and Parahuleva, 2025; Wang et al., 2025).

Numerous studies have investigated alterations in microRNA expression in human carotid atherosclerotic lesions using various groups of comparison patient cohorts and different experimental approaches (Cipo et al., 2011; Lovren et al., 2012; Nazari-Jahantigh et al., 2012; Cao et al., 2014; Bazan et al., 2015; Maitrias et al., 2015; Markus et al., 2016; Jin et al., 2018; Katano et al., 2018; Wang et al., 2019; Collura et al., 2021; González-López et al., 2022; Nazarenko et al., 2022; Deng et al., 2023). Although the reported findings vary considerably and only partially overlap, by the end of 2024, more than two independent studies had demonstrated altered expression of miR-21, miR-143/-145, miR-100, and miR-127 during the development and destabilization of carotid atherosclerotic plaques (Koroleva et al., 2017; Jin et al., 2018; Katano et al., 2018; Wang et al., 2019; González-López et al., 2022; Nazarenko et al., 2022).

MicroRNA expression is regulated by multiple mechanisms, including epigenetic modifications such as DNA methylation of regulatory genomic regions. Several types of regulatory elements may contribute to methylation-dependent regulation of the microRNA genes, including (Aavik et al., 2015): promoter regions enriched in CpG sites capable of undergoing methylation (Ali Syeda et al., 2020); CpG islands located in the proximal promoter regions of microRNA gene (Aryee et al., 2014); CpG islands upstream of transcription start site (TSS) of microRNA cluster (Baer et al., 2013); CpG island located in the promoter of the host gene of intronic microRNA (Bazan et al., 2015); distant enhancers that can regulate microRNA expression (Morales et al., 2017).

Most studies investigating DNA methylation of microRNA regulatory regions have focused on cancer. These studies have demonstrated that hypermethylation of promoter-associated CpG islands is commonly associated with reduced microRNA expression, whereas promoter or enhancer hypomethylation may result in transcriptional activation (Baer et al., 2013; Chhabra, 2015; Bell et al., 2016; Morales et al., 2017; Ali Syeda et al., 2020; Saviana et al., 2023). In contrast, the contribution of DNA methylation of microRNA genes to other common diseases, including atherosclerosis, remains poorly understood (Ek et al., 2016; Sharma et al., 2020; Cui et al., 2024).

Epigenome-wide association studies (EWAS) typically examine DNA methylation at CpG sites within an intergenic region or inside protein-coding genes in peripheral blood cells from patients with different manifestations of atherosclerosis (Istas et al., 2017; Liu et al., 2017; Soriano-Tárraga et al., 2018; Sharma et al., 2020; Ingold et al., 2026). Comparatively few studies have investigated DNA methylation changes in vascular tissues or atherosclerotic plaques (Castillo et al., 2010; Fernandez et al., 2011; Zaina et al., 2014; Aavik et al., 2015; Nazarenko et al., 2015; Zaina et al., 2015; Li et al., 2021). Previous studies comparing advanced atherosclerotic and intact vascular tissues have reported both decreased DNA methylation in genomic regions encompassing *MIR10B* and *MIR127* (Aavik et al., 2015; Nazarenko et al., 2015) and increased DNA methylation within the *MIR143*/*MIR145* cluster (Ehrlich et al., 2019).

To date, no studies have comprehensively examined DNA methylation level of regulatory elements of microRNA genes in carotid atherosclerotic plaques. Because DNA methylation represents a potential mechanism regulating microRNA expression, characterization of DNA methylation patterns within both regulatory regions and gene bodies of microRNA genes associated with plaque instability is warranted in carotid atherosclerotic plaques and peripheral blood leukocytes from patients with carotid atherosclerosis. Identifying disease-associated DNA methylation patterns in vascular tissues and blood may improve our understanding of the epigenetic mechanisms underlying advanced atherosclerosis and provide a basis for further investigation of DNA methylation markers for plaque instability and atherosclerosis-related complications.

## 2 Methods

### 2.1 Study participants and sample collection

This study included 92 patients with advanced carotid atherosclerosis (70% men, median age 64 years) and 32 subclinical atherosclerosis controls (60% men, median age 67 years) (Supplementary Material). All participants underwent carotid ultrasonography. The patient group comprised individuals with severe carotid stenosis (>65%), an indication for carotid endarterectomy. Control participants had early-stage carotid atherosclerosis without hemodynamically significant changes: the degree of stenosis was 28 [24; 45]% (M [Q1; Q3]), and intima-media complex thickness was 0.8 [0.7; 0.9] mm. Peripheral blood samples were collected from each control participant (PBC). The study was conducted in accordance with the Declaration of Helsinki and approved by the Local Biomedical Ethics Committee at the Research Institute of Medical Genetics, Tomsk National Research Medical Center (protocol #4, 25 November 2019).

Peripheral blood samples were collected from each patient before carotid endarterectomy (PBP; n = 92). During surgery, carotid atherosclerotic plaque samples (ASP; n = 92), macroscopically intact adjacent carotid artery tissue (ICA; n = 34), and great saphenous vein samples (GSV; n = 23) were obtained. Each ASP sample was divided into 3 equal fragments with similar macroscopic characteristics. One fragment was used for histological verification of the ASP type according to the American Heart Association (AHA) system (Stary et al., 1995), assessment of plaque stability, and evaluation of pathomorphological characteristics (Supplementary Material). A second fragment was used for DNA extraction, sodium bisulfite treatment, and DNA methylation analysis. The third fragment was placed back into a tube containing a reagent for RNA stabilization and sample storage in RNALater (Dia-m, Russia) for subsequent analysis.

### 2.2 DNA methylation analysis in vascular tissues and peripheral blood

We selected microRNAs (miR-10b, miR-21, miR-100, miR-127, miR-143 and miR-145) whose expression was associated with impaired stability of atherosclerotic plaques, according to the literature (Supplementary Table 4). For the literature search, we used publications indexed in HMDD v3.2: the Human miRNA Disease Database (http://www.cuilab.cn/hmdd) (Cui et al., 2024) and NCBI: National Center for Biotechnology Information (https://www.ncbi.nlm.nih.gov).

To identify the genomic locations of regulatory elements of genes and/or host genes of selected microRNAs, we used FANTOM5 (http://fantom.gsc.riken.jp/5/) (De Rie et al., 2017) and TRmir databases (Gao et al., 2022), as well as publications from the aforementioned HMDD and NCBI databases.

Target regions for the methylation level analysis were successfully selected for *MIR10B, MIR21, MIR127* and *MIR143* genes (Supplementary Table SM5). Additionally, the *MIR10B* gene was included in the study to validate and extend the findings from our laboratory’s previous research (Nazarenko et al., 2015).

DNA methylation at CpG sites in the target regions was quantified using Bisulfite Amplicon Sequencing (BSAS) performed on the MiSeq platform (Illumina, United States) using massive parallel sequencing (Masser et al., 2015). All the BSAS stages, as well as the target region selection procedure, and genomic coordinates of the analyzed CpG sites are described in Supplementary Material.

To compare our findings with external data on microRNA gene methylation in vascular tissues and blood cells, we selected five publicly available DNA methylation datasets from the Gene Expression Omnibus (GEO) data repository (https://www.ncbi.nlm.nih.gov/geo): GSE46394 (Zaina et al., 2014), GSE66500 (Zaina et al., 2015), GSE149759 (Li et al., 2021), GSE69138 (Soriano-Tárraga et al., 2018), and GSE246337 (Moqri et al., 2026) (Supplementary Table S11).

### 2.3 Lab-internal DNA methylation and microRNA expression analysis in atherosclerotic plaques

To examine methylation at CpG sites associated with miRNA genes using microarray data, we analyzed lab-internal EWAS dataset of DNA methylation and microRNA expression in ASP samples. A total of 16 ASP samples were selected according to histological stability and divided into two equal groups: 8 type III-V plaques (preatheroma and stable lesions) and 8 type VI plaques (complicated lesions), based on the AHA classification (Supplementary Material - Supplementary Methods). DNA and RNA were extracted from fragments of the same carotid artery samples from 16 patients – 10 men and 6 women, with a median age of 65 years (Supplementary Material - Supplementary Methods).

DNA methylation was profiled using the Illumina Infinium MethylationEPIC BeadChip microarray according to the manufacturer’s instructions. For microRNA expression analysis by miRNA-seq, cDNA libraries were prepared using the NEBNext Multiplex Small RNA Library Prep Set for Illumina (New England Biolabs, United States) and sequenced on the HiSeq 1,500 (Illumina, United States). Sequencing was performed in single-end mode with a read length of 50 nucleotides. All the stages of microRNA expression analysis are detailed in Supplementary Material - Supplementary Methods.

### 2.4 Data processing and statistical analysis

BSAS data were processed using established bioinformatic methods and pipelines (Supplementary Material - Supplementary Methods). For each CpG site, the methylation level was expressed as beta (β) value, calculated as the percentage of reads corresponding to methylated cytosine among all cytosine reads at a specific genomic position.

For the lab-internal EWAS, microarray signal intensities were scanned using an iScan® System scanner (Illumina, United States) and recorded as images and raw IDAT files. These data were used to analyze the level of DNA methylation using Bioconductor packages (Aryee et al., 2014) in the R, free software environment for statistical computing and graphics (R Core Team, 2025). The data on the CpG sites of microRNA genes provided in the annotation of the microarray manufacturer (Illumina, United States) have been updated in accordance with the human genome version GRCh37/hg19 and supplemented with information on miRNA gene regulatory elements from the TRmir database (Gao et al., 2022).

MicroRNA expression was quantified using the miARma-Seq pipeline, with reads subjected to quality control and adapter trimming, aligned to the GRCh37/hg19 reference genome, and normalized as counts per million (CPM). The information regarding miRNA primary transcripts and mature microRNAs (miRNA) was obtained from the miRBase v.22.1 (Kozomara et al., 2019).

To assess sequencing-depth-associated uncertainty in DNA methylation measurements, a Monte Carlo simulation was performed using CpG-specific methylation percentages and sequencing coverage. CpG sites with a coverage below 50 reads were excluded. For each remaining CpG, 10,000 methylation values were simulated using a binomial model based on the observed methylation proportion and coverage. Regional methylation was calculated as the unweighted mean across CpG sites for each simulation. The 95% Monte Carlo confidence intervals were used to quantify measurement uncertainty associated with sequencing depth. This analysis served to estimate coverage-derived variance rather than total assay-specific technical variation.

Statistical analyses were performed in R (version 4.5.2 and earlier versions) using standard statistical packages (R Core Team, 2025). CpG site methylation levels are reported as M [Q1; Q3], where M denotes the median and Q1 and Q3 denote the first (25th percentile) and third (75th percentile) quartiles, respectively.

Differences in DNA methylation between study groups were assessed using nonparametric tests. The Wilcoxon signed-rank test was used for paired comparisons of different tissue types obtained from the same patients, whereas the Mann-Whitney U test was used for independent comparisons of peripheral blood leukocytes from patients and controls. Correlations were assessed using Spearman’s rank correlation coefficient, and correlation magnitudes were interpreted according to Schober et al. (2018).

Potential demographic and clinical covariates were included in the statistical analyses where appropriate, depending on their availability and relevance to specific study comparisons.

A nominal *p* value <0.05 was considered statistically significant. To account for multiple testing, *p* values were adjusted using the Benjamini–Hochberg procedure to control the false discovery rate (FDR) within each analytical comparison (Benjamini and Hochberg, 1995). Ultimately, only FDR-adjusted *p* values were used to define statistically significant associations.

### 2.5 Estimation of cell-type fractions in vascular tissues and peripheral blood

Cell-type fractions in vascular tissues and peripheral blood were estimated using two categories of DNA methylation (DNAm) datasets: external microarray datasets (GSE46394, GSE66500, GSE149759, and GSE246337) and the lab-internal EWAS dataset described above. External datasets were downloaded from GEO as normalized beta values.

For vascular tissues, the proportions of major cell populations were estimated using the original DNAm reference matrices (Supplementary Table S12) in combination with the HEpiDISH algorithm (Teschendorff et al., 2017; Zheng et al., 2018). The analysis included vascular endothelial cells (VECs), smooth muscle cells (SMCs), fibroblasts (FIB), and immune cells (ICs), including B cells, natural killer (NK) cells, CD4^+^ and CD8^+^ T cells, monocytes (Mono), neutrophils (Neutro), and eosinophils (Eosino) (Supplementary Table S13). Associations between the estimated cell-type proportions and methylation levels at 28 miRNA-associated CpG sites annotated on the Illumina platform (Supplementary Table S10) were assessed using Weighted Gene Co-expression Network Analysis (WGCNA) (Langfelder and Horvath, 2012). Based on these analyses, CpG sites showing the strongest associations with SMCs and ICs were selected to construct a model applicable to the targeted bisulfite sequencing data. This model was used to determine whether variation in microRNA gene methylation was primarily driven by changes in the functional state of vascular cells during carotid artery pathology or by shifts in cell-type composition.

For peripheral blood we chose one DNA methylation (DNAm) reference matrix designed for adult whole blood in EpiDISH algorithm (Teschendorff et al., 2017). Blood cell-type estimation in the external EWAS dataset GSE246337 was evaluated using the same 28 miRNA-associated CpG sites on Illumina methylation microarrays.

## 3 Results

### 3.1 Tissue specificity of DNA methylation level of *MIR10B*, *MIR21*, *MIR100*, *MIR127* and *MIR143* gene regions between blood and vascular tissues

We analyzed DNA methylation in eight regions related to five microRNA genes.

1. The CpG island overlaying *MIR10B* gene body; 23 CpG sites (hereafter referred to as *MIR10B* region);
2. The literature-defined putative promoter of *MIR21* gene; 9 CpG sites (*MIR21* putative promoter);
3. The *MIR21* CpG island described by Iorio et al. (2007); 16 CpG sites;
4. The *MIR21* gene body; 5 CpG sites;
5. The E-box region containing the transcription factor ZEB1 binding site and representing the putative regulatory region of *MIR100* gene (Chen et al., 2014); 7 CpG sites (*MIR100* E-box region);
6. The CpG island overlapping with *MIR127* gene body; 40 CpG sites (*MIR127* region);
7. The *MIR143HG* promoter, representing the regulatory region of *MIR143* gene; 10 CpG sites;
8. The *MIR143* gene body; 5 CpG sites.

For the intergenic miRNA genes *MIR10B* and *MIR127*, the selected regions comprised CpG islands that fully or partially overlapped the gene bodies. For the intragenic genes *MIR21* and *MIR143*, the gene bodies and putative regulatory regions were analyzed, including the MIR21 CpG island.

Across all eight regions, median DNA methylation patterns were tissue-specific. Methylation profiles were highly similar between peripheral blood leukocytes from patients (PBP) and controls (PBC) but differed between blood leukocytes and vascular tissues, including carotid atherosclerotic plaques (ASP), macroscopically intact adjacent carotid artery tissue (ICA), and great saphenous veins tissue (GSV) (Figures 1, 2; Supplementary Tables S01-S09).

**FIGURE 1:**
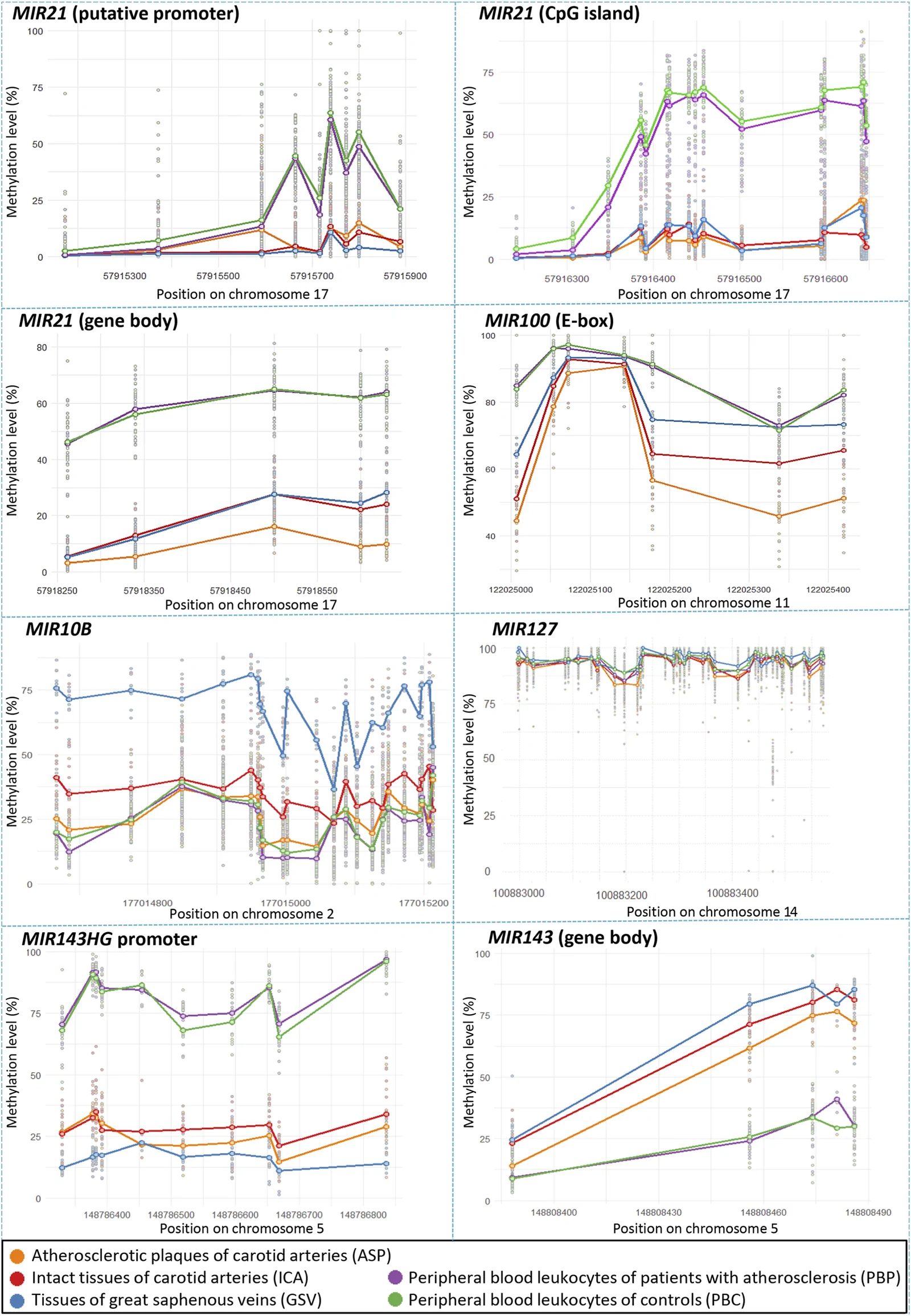
DNA methylation profiles across the investigated microRNA gene regions. Genomic coordinates are given according to the GRCh37/hg19 genome assembly. Each point represents an individual CpG methylation value, and lines connect the median methylation levels within each tissue. Note: Axis ranges are adjusted for each region to improve visualization of within-region differences; therefore, absolute methylation levels should not be compared visually across panels.

**FIGURE 2:**
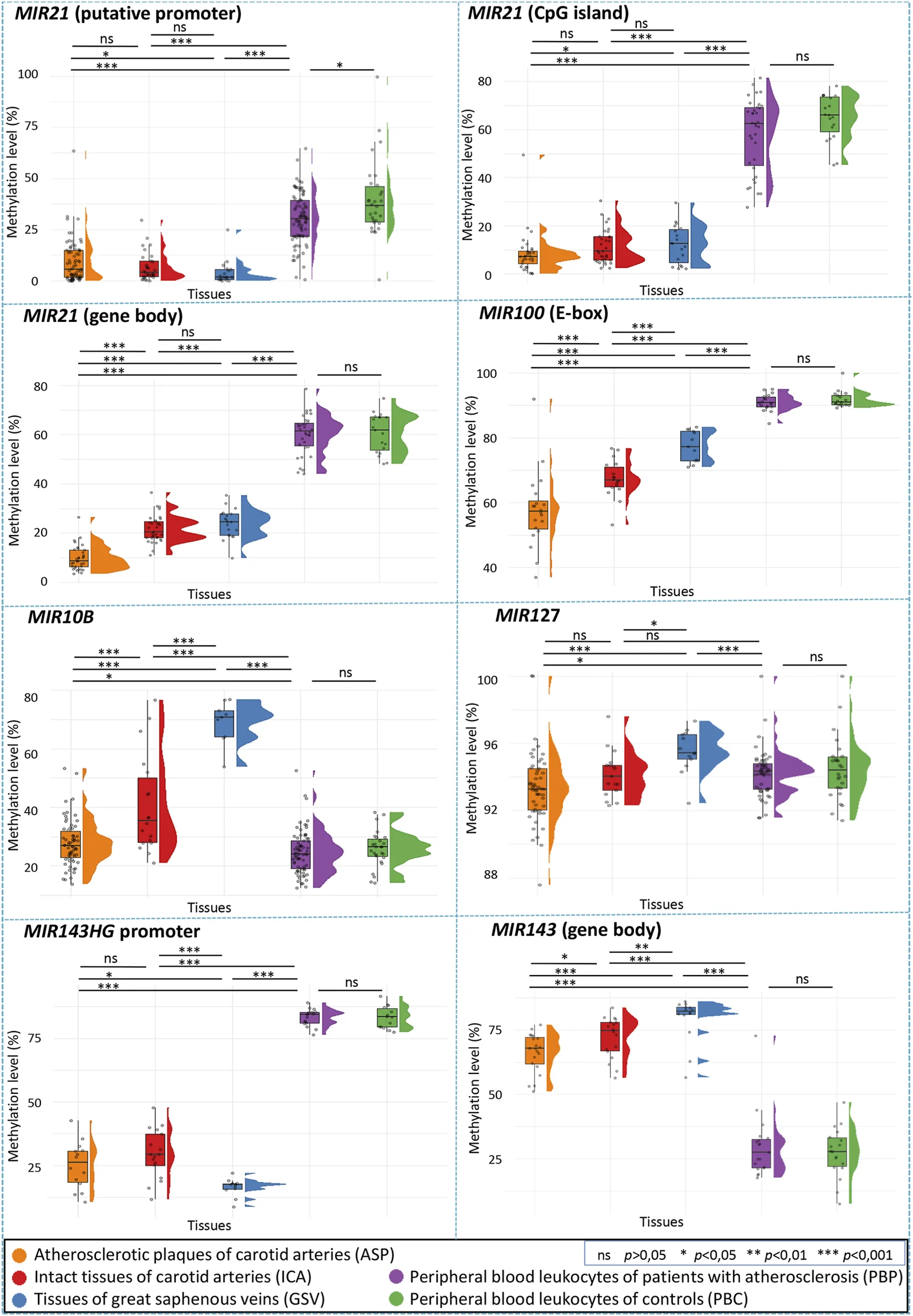
Comparison of median DNA methylation across the investigated microRNA gene regions. Each point represents the median methylation level across all CpG sites within the corresponding region for an individual sample. Note: Axis ranges are adjusted for each region to improve visualization of within-region differences; therefore, absolute methylation levels should not be compared visually across panels.

In the *MIR127* region, DNA methylation levels were generally high (80%–100%) across all tissues (in Figure 1, the upward shift of the plots for *MIR127* should be noted). However, a distinct cluster of outlying samples with approximately 50% methylation was consistently observed at CpG site chr14:101349764 (here and further, GRCh37/hg19 coordinates). This CpG overlaps with the single nucleotide variant rs34163305:C>T, for which the minor T allele has a frequency of 0.24 in the European population according to gnomAD v.4.1.0 database (https://gnomad.broadinstitute.org/variant/14-100883427-C-T). Because the T allele abolishes the CpG dinucleotide, methylation cannot occur in TT homozygotes, whereas values of approximately 50% are expected in heterozygotes.

Median methylation was higher in leukocytes (PBP and PBC) than in vascular tissues (ASP, ICA, and GSV) at all three *MIR21* regions: the putative promoter (min-max: 30.3%–36.8% vs. 1.7%–5.7%), CpG island (min-max: 62.4%–66.1% vs. 7.2%–12.7%) and gene body (min-max: 61.4%–61.8% vs. 8.7%–24.5%). Similarly elevated methylation in leukocytes was observed in the *MIR100* E-box region (min-max: 90.9%–91.0% vs. 57.3%–77.2%) and *MIR143HG* promoter (min-max: 83.5%–84.4% vs. 17.4%–29.3%) (Figure 2; Supplementary Tables S01-S09).

By contrast, median methylation of the *MIR143* gene body was lower in PBP and PBC than in vascular tissues (min-max: 27.5%–27.7% vs. 67.8%–82.2%, *p* < 0.001). Median methylation of the *MIR10B* region was also lower in leukocytes than in GSV (min-max: 24.0%–26.5% vs. 70.8%) and carotid tissues (ASP and ICA; min-max 26.9%–35.4%) (Figure 2). However, these differences at individual CpG sites were small or not statistically significant (Figure 1; Supplementary Tables S01-S09). Similar to *MIR10B* region, the highest median methylation in *MIR127* region was detected in GSV (Figure 2; Supplementary Table S07).

Although several comparisons reached statistical significance, the magnitude of these differences was small, not exceeding 2.2%. To assess the extent to which the observed variation could be attributed to sequencing-depth-associated measurement uncertainty, we additionally performed a Monte Carlo analysis based on CpG-specific sequencing coverage. The 95% Monte Carlo confidence intervals for regional methylation were generally below ±1 percentage point (0.08–0.67 percentage points) after excluding CpG sites with coverage <50 reads. Thus, the larger observed differences between tissues were unlikely to be explained solely by coverage-derived measurement variability.

### 3.2 Methylation of microRNA genes in vascular tissues of patients with carotid artery atherosclerosis

#### 3.2.1 CpG islands of *MIR10B* and *MIR127* genes, gene bodies of *MIR21* and *MIR143*, and *MIR100* regulatory region are moderately hypomethylated in carotid atherosclerotic lesions

Compared with ICA, ASP demonstrated significantly lower median DNA methylation (Figure 2; Supplementary Tables S01-S09) in the *MIR10B* region (by 8.5%, *p* < 0.001), *MIR21* gene body (by 11.7%, *p* < 0.001), *MIR100* E-box region (by 9.7%, *p* < 0.001) and *MIR143* gene body (by 6.9%, *p* = 0.019). Median methylation in these regions was also lower in ASP than in GSV; the same pattern was observed for *MIR127* region (Figure 2; Supplementary Tables S01-S09).

To validate these findings, our BSAS results were compared with the lab-internal EWAS dataset and publicly available EWAS datasets from GEO (the genomic correspondence between BSAS target regions and Illumina methylation microarrays probes is summarized in Supplementary Table S10. Notably, the *MIR100* E-box region does not contain CpG sites annotated on the Illumina microarray platform).

Methylation patterns for *MIR10B*, *MIR21* and *MIR143* were highly consistent across independent studies, analytical platforms, and vascular tissues, except for the *MIR21* gene body in the internal mammary artery (IMA) samples from GSE149759 (Figure 3). In contrast, methylation of cg21242144 in the *MIR143* gene body differed substantially between BSAS and microarray datasets.

**FIGURE 3:**
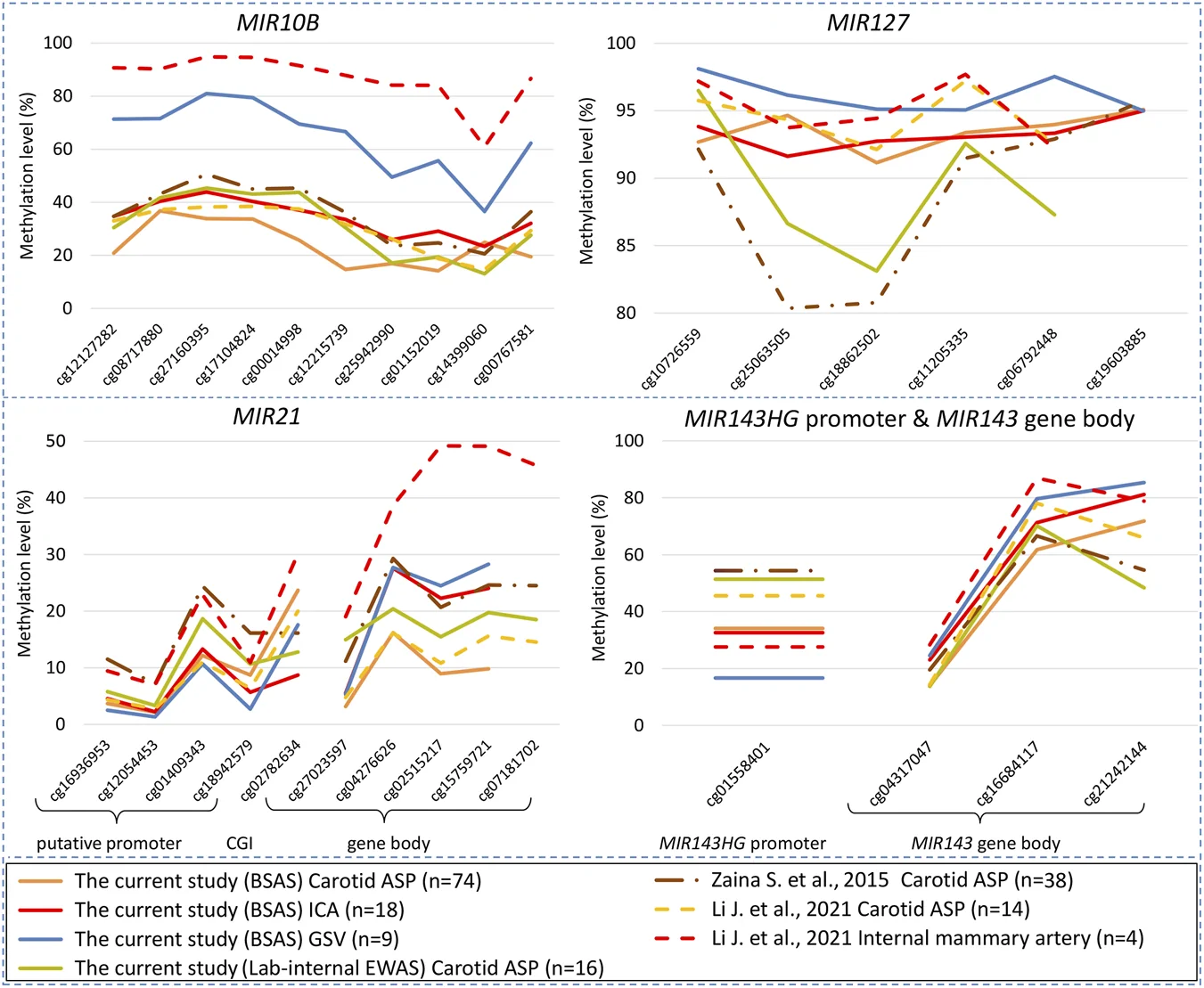
CpG methylation patterns in different blood vessels and carotid plaques measured by bisulfite amplicon sequencing (BSAS) and Illumina methylation microarrays technology (external and lab-internal EWAS). ASP, carotid atherosclerotic plaques; ICA, intact carotid artery; GSV, great saphenous veins. Note: Axis ranges are adjusted for each region to improve visualization of within-region differences; therefore, absolute methylation levels should not be compared visually across panels.

The methylation profile of ASP samples in lab-internal EWAS was similar to that reported by Zaina et al. (2015), whereas greater discrepancies were observed relative to the dataset published by Li et al. (2021). Because all three studies used Illumina methylation arrays, these inconsistencies are likely attributable to biological rather than technical factors, including differences in patient age, ethnicity, vascular cell composition, and disease characteristics.

Across all CpG sites in the *MIR10B* region, *MIR21* gene body and *MIR143* gene body, methylation was lower in ASP than in IMA or GSV in BSAS and in microarray datasets (Figure 3) (Zaina et al., 2015; Li et al., 2021; lab-internal EWAS (unpublished data)). In contrast, *MIR127* methylation was uniformly high (>80%) across all vascular tissues, without a clear disease-specific methylation pattern. Nevertheless, analysis of BSAS data separately suggested lower methylation in ASP than in GSV (Figure 3).

Unlike the other analyzed regions, the *MIR143HG* promoter, represented by single cg01558401 site, exhibited higher DNA methylation in ASP than in unaffected vessels (min-max: 34.2%–54.4% vs. 16.7%–32.6%) (Figure 3) (Zaina et al., 2015; Li et al., 2021; lab-internal EWAS (unpublished data)).

#### 3.2.2 *MIR100* and *MIR127* gene methylation remains associated with carotid atherosclerosis after adjustment for estimated vascular cell composition

Cellular heterogeneity is an important potential confounding factor in DNA methylation studies of vascular tissues because blood vessels contain variable proportions of stromal and immune cell populations, each characterized by distinct DNA methylation profiles. Consequently, observed methylation differences reflect shifts in cellular composition rather than providing accurate insights into the trait being investigated (Eipel et al., 2016; Berg et al., 2025).

To account for this source of variation, cell-type proportions were estimated using data from 102 vascular samples derived from combined external EWAS datasets (GSE149759, GSE46394, GSE66500) and lab-internal EWAS (Supplementary Table S11). Because SMCs, VECs, and ICs represent major cellular populations that may vary during atherosclerotic plaque progression, their estimated proportions were included as potential confounders in subsequent regression analyses. These estimates are computational rather than direct measurements of cell abundance, and the deconvolution approach cannot completely exclude residual confounding by cellular heterogeneity.

The deconvolution analysis revealed distinct associations between methylation at miRNA-associated CpG sites and estimated vascular cell proportions. *MIR10B*, *MIR127* (except cg25063505), and *MIR143* gene body methylation showed positive associations with the estimated SMC proportion, whereas methylation in the *MIR21* and *MIR143HG* promoter regions was positively associated with the estimated IC proportion. The strongest positive association was observed between methylation of cg01558401 in the *MIR143HG* promoter and the estimated ICs proportion (*rho* = 0.96, *p* < 0.001) (Figure 4). Two CpG sites in the *MIR143* gene body (cg16684117 and cg21242144) showed similarly strong positive correlations with the estimated SMC proportion (*rho* = 0.92 and 0.91 respectively) (Figure 4).

**FIGURE 4:**
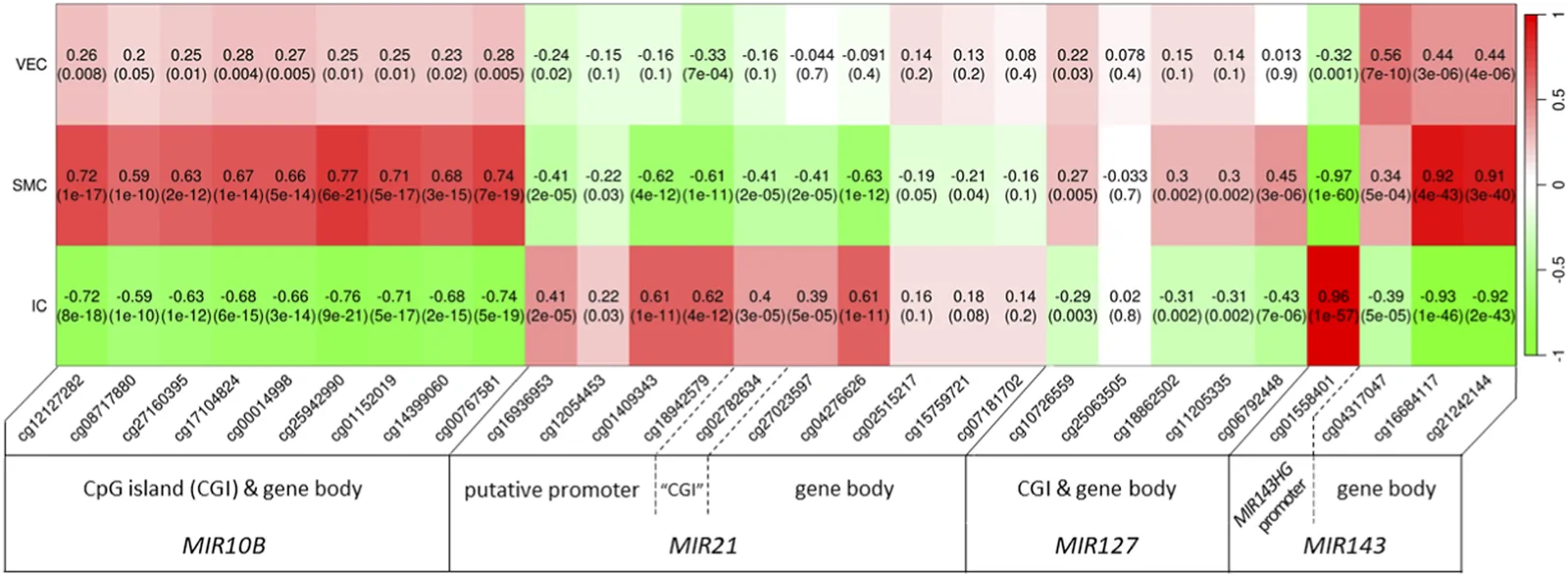
Correlation heatmap between DNA methylation of analyzed CpG sites and estimated proportions of vascular endothelial cells (VECs), smooth muscle cells (SMCs) and immune cells (ICs).

Based on these associations, cg01558401 in the *MIR143HG* promoter and cg21242144 in the *MIR143* gene body were selected for further analysis because they showed the strongest associations with the estimated IC and SMC proportions, respectively, and were also covered by BSAS.

As expected, the opposite relationships of these CpG sites with the estimated IC and SMC proportions resulted in a strong inverse correlation between their methylation levels. This relationship was observed consistently in the combined EWAS datasets (*rho* = −0.801, *p* < 0.001) (Figure 5A) and independently confirmed in the BSAS cohort (*rho* = −0.784, *p* < 0.001) (Figure 5B).

**FIGURE 5:**
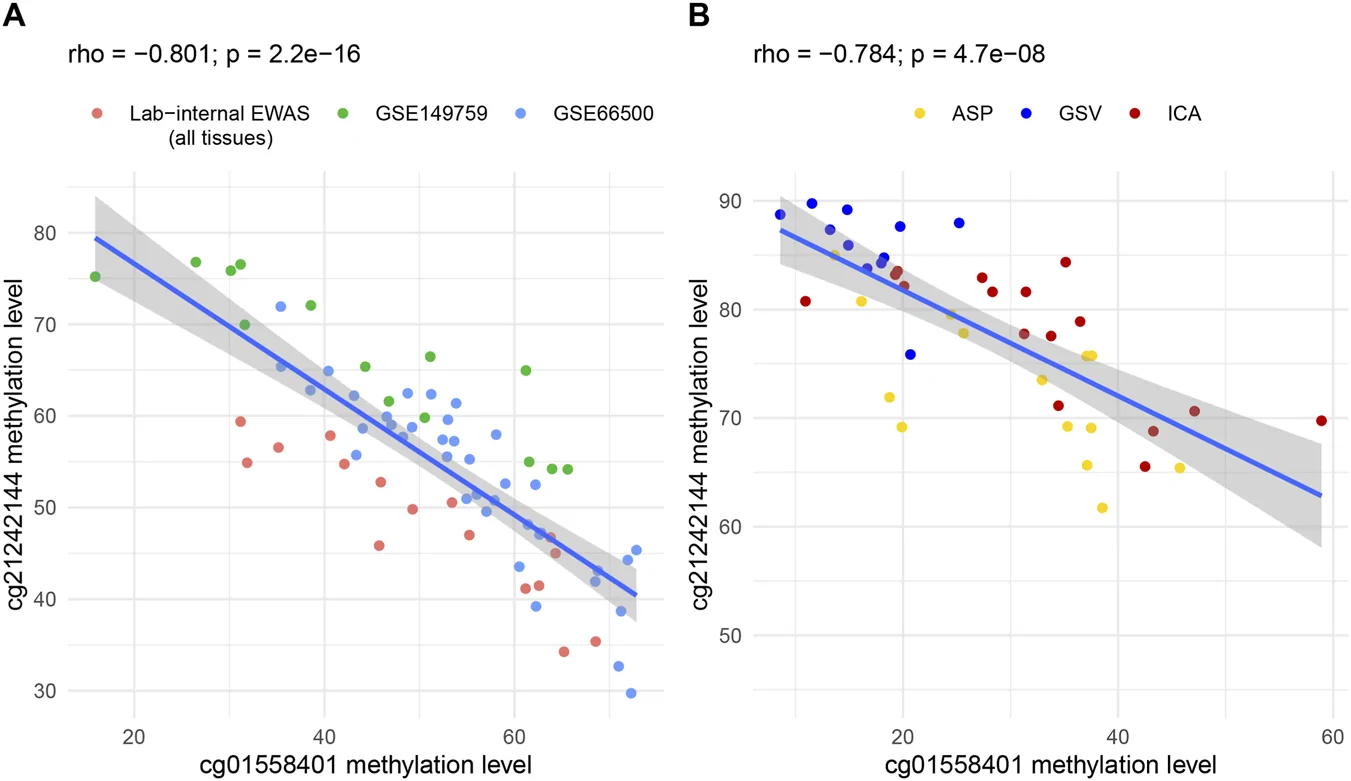
Correlation between DNA methylation of cg01558401 (in *MIR143HG* promoter) and cg21242144 (in *MIR143* gene body) in the combined EWAS datasets **(A)** and BSAS cohort **(B)**.

To exclude age as a potential confounder, we examined the relationship between methylation at these CpG sites and patient age. No significant associations were detected (cg01558401: *rho* = −0.091, *p* = 0.604; cg21242144: *rho* = 0.113, *p* = 0.519), indicating that methylation of two target CpG sites remains stable within the studied age range (58–70 years).

Linear models were subsequently generated to estimate the proportion of SMCs/ICs based on BSAS methylation values of cg01558401 and cg21242144. The intercepts and coefficients obtained from these models were used to formulate the estimation equations for SMC and IC proportions.

Estimated % of Smooth Muscle Cells=0.6266−0.9531×cg01558401 methylation+0.5454×cg21242144 methylation

Estimated % of Immune Cells=0.3856+0.9102×cg01558401 methylation−0.6877×cg21242144 methylation

Finally, regression models were fitted to the targeted BSAS data to evaluate associations of CpG methylation with carotid artery state (ICA or ASP) and estimated SMC or IC proportion (Table 1). This analysis aimed to determine whether DNA methylation differences reflected changes in vascular cell composition or disease-associated functional alterations in carotid cells.

**TABLE 1:** Regression analysis of CpG sites in microRNA genes methylation levels in relation to carotid artery state and the estimated proportion of vascular cells.

Effects	Estimate	Std. Error	t value	Pr (>|t|)	*p(FDR)*	CpG	microRNA gene
Intercept	0.733	0.239	3.071	0.004	0.039	chr11:122025072	*MIR100* (E-box region)
ASP	−0.066	0.021	−3.161	0.003	0.032		
Intercept	1.262	0.338	3.733	0.001	0.009	*chr11:122025178*	
ASP	−0.264	0.029	−8.956	1.08E-10	1.08E-10		
ICA	−0.149	0.030	−4.968	1.66E-05	3.83E-04		
Intercept	1.446	0.374	3.867	4.43E-04	0.006	*chr11:122025419*	
ASP	−0.309	0.033	−9.456	2.71E-10	5.41E-09		
ICA	−0.176	0.033	−5.301	5.97E-06	1.63E-04		
Intercept	1.262	0.180	7.023	4.18E-08	2.51E-06	*chr14:101349589*	*MIR127*
ASP	−0.053	0.016	−3.295	0.002	0.026		
ICA	−0.052	0.016	−3.193	0.003	0.031		
Intercept	1.127	0.172	6.536	1.75E-07	6.56E-06	*chr14:101349597*	
ASP	−0.049	0.015	−3.197	0.003	0.031		
ICA	−0.046	0.015	−2.970	0.005	0.049		
Intercept	1.146	0.112	10.279	2.96E-12	8.89E-10	**cg01558401** **(chr5:148786377)**	*MIR143HG* promoter
ASP	−0.031	0.010	−3.219	0.003	0.029		
SMC	−1.048	0.118	−8.854	1.45E-10	1.24E-08		
Intercept	1.122	0.218	5.141	9.75E-06	2.44E-04	chr5:148786392	
SMC	−1.027	0.232	−4.432	8.41E-05	0.001		
Intercept	1.106	0.222	4.985	2.07E-05	4.44E-04	cg16684117 (chr5:148808456)	*MIR143* gene body
ASP	−0.110	0.020	−5.476	4.97E-06	1.42E-04		
IC	−0.764	0.247	−3.087	0.004	0.039		
Intercept	0.727	0.196	3.710	0.001	0.010	**cg21242144** **(chr5:148808486)**	
ASP	−0.056	0.017	−3.286	0.002	0.026		

Std. error, standard error of the estimate; Pr (>|t|), *p*-value for the t-test; *p* (FDR), FDR-adjusted *p*-values. ASP, atherosclerotic plaque; ICA, intact carotid artery; SMC, smooth muscle cells; IC, immune cells. Models were adjusted for all covariates. CpG sites used to generate the model for estimation are shown in **bold**. CpG sites influenced by *both* carotid atherosclerosis (ASP) and normal artery state (ICA) – that is, rather by functional changes in carotid artery cells – but not by estimated proportion of smooth muscle cells (SMCs) or immune cells (ICs) are shown in *italics*.

In these models, methylation at four CpG sites, two in the *MIR100* E-box region and two in the *MIR127* region, remained significantly associated with carotid artery state after adjustment for estimated SMC or IC proportions. These findings suggest that methylation differences at these sites are not fully accounted for by vascular cell composition and may therefore reflect atherosclerosis-associated changes in the carotid artery tissue.

In contrast, methylation at several other CpG sites was associated with carotid artery state (ASP) and/or estimated cell proportions (Table 1), suggesting that this variability may reflect contributions from both disease-associated tissue differences and cellular composition.

#### 3.2.3 DNA methylation levels of the *MIR10B*, *MIR21*, and *MIR127* genes in carotid atherosclerotic plaque are associated with indicators of histological plaque instability and acute cerebrovascular events

We investigated the associations between the DNA methylation levels across all analyzed microRNA gene regions in carotid ASP and both qualitative and quantitative indicators of histological plaque instability.

Within the *MIR21* putative promoter, methylation levels of three CpG sites (chr17:57915593, cg12054453 and cg18942579), as well as the median methylation across the entire region, were significantly lower in ASPs with intraplaque hemorrhage in the plaque shoulder than in ASP without hemorrhages (Table 2). Similarly, five of the nine analyzed CpG sites, including the three sites mentioned above, together with the median methylation across the region, showed significantly reduced methylation in ASPs containing foam cells (Table 2).

**TABLE 2:** Statistically significant changes of methylation level in CpG sites and entire microRNA genes regions depending on individual qualitative indicators of the atherosclerotic plaque histological instability.

Region/CpG site	β if present	β if absent	Δβ	β if present	β if absent	Δβ
*MIR10B*	Thrombus on the plaque shoulder surface			​		
	n = 7	n = 35	​			
cg14399060 (chr2:177015070)	8.6 [8.3; 10.7]%	28.6 [21.8; 38.0]%	−20.0 (*p* < 0.001)			
*MIR21* putative promoter	Hemorrhages in the plaque shoulder			Presence of foam cells		
	n = 18	n = 26	​	n = 16	n = 28	​
Median	1.6 [0.8; 6.7]%	8.9 [3.2; 14.8]%	−7.3 (*p* = 0.019)	1.8 [1.3; 3.6]%	8.9 [3.1; 15.1]%	−7.1 (*p* = 0.032)
chr17:57915172	*non-significant*			0.1 [0.0; 0.3]%	0.6 [0.2; 3.3]%	−0.5 (*p* = 0.032)
chr17:57915593	0.7 [0.0; 15.8]%	17.9 [5.2; 37,4]%	−17.6 (*p* = 0.019)	1.2 [0.0; 7.3]%	22.7 [9.0; 36.7]%	−21.5 (*p* = 0.032)
cg12054453 (chr17:57915717)	0.6 [0.2; 2.3]%	2.5 [1.1; 8,7]%	−1.9 (*p* = 0.019)	0.8 [0.2; 1.7]%	2.6 [1.1; 8.5]%	−1.8 (*p* = 0.049)
cg18942579 (chr17:57915773)	4.0 [1.2; 8.6]%	13.9 [6.3; 24.6]%	−9.9 (*p* = 0.019)	2.6 [1.5; 8.8]%	13.7 [6.0; 22.4]%	−11.1 (*p* = 0.032)
chr17:57915800	*non-significant*			8.3 [4.8; 17.1]%	17.0 [9.0; 28.9]%	−8.7 (*p* = 0.049)
*MIR21* gene body	Calcifications in the plaque shoulder			Fibrous cap rupture		
	n = 5	n = 10	​	n = 8	n = 7	​
Median	9.3 [8.8; 10.3]%	6.8 [5.6; 8.8]%	2.5 (*p* = 0.044)	9.3 [7.6; 10.9]%	6.3 [5.3; 8.3]%	3.0 (*p* = 0.044)
cg02515217 (chr17:57918601)	11.0 [9.0; 13.0]%	6.2 [5.1; 7.4]%	4.8 (*p* = 0.044)	9.4 [5.7; 13.3]%	6.7 [5.8; 7.8]%	2.7 (*p* = 0.044)

β, methylation level; Δβ, difference in methylation level between groups; n, sample size. *p,* adjusted level of significance (FDR-corrected).

In contrast, methylation of cg02515217 and the median methylation of the *MIR21* gene body were significantly higher in plaques with fibrous cap rupture or calcification in the plaque shoulder. We also observed significantly lower methylation of cg14399060 in the *MIR10B* region in plaques with thrombus on the plaque shoulder surface (Table 2).

We also identified moderate to strong correlations (|*rho*| = 0.4.0.9) between methylation levels at individual CpG sites in the ASPs and such quantitative histological indicators of atherosclerotic plaque instability as the number of inflammatory cells per field of view (Table 3).

**TABLE 3:** Moderate and strong correlations of methylation level of individual CpG sites in atherosclerotic plaque with individual quantitative histological indicators of plaque instability.

Quantitative histological indicators of plaque instability	Region	CpG	Correlation coefficient and level of significance
Cap thickness, microm	*MIR127*	**chr14:101349509**	*rho* = −0.450; *p* = 0.016
		chr14:101349515	*rho* = −0.510; *p* = 0.008
Size of necrotic core, microm	*MIR127*	cg25063505 (chr14:101349286)	*rho* = −0.424; *p* = 0.035
		**chr14:101349509**	*rho* = −0.443; *p* = 0.018
		chr14:101349718	*rho* = −0.520; *p* = 0.002
Number of neutrophils (per field of view)	*MIR127*	chr14:101349561	*rho* = *−0.767; p* = *0.010*
		chr14:101349718	*rho* = −0.673; *p* = 0.033
Number of lymphocytes (per field of view)	*MIR127*	**chr14:101349643**	*rho* = −0.502; *p* = 0.006
		**chr14:101349784**	*rho* = 0.405; *p* = 0.018
Number of macrophages (per field of view)	*MIR127*	**chr14:101349643**	*rho* = −0.571; *p* = 0.005
	*MIR127*	**chr14:101349784**	*rho* = 0.410; *p* = 0.037
Number of foam cells (per field of view)	*MIR10B*	chr2:177015172	*rho* = 0.538; *p* = 0.032
		chr2:177015208	*rho* = 0.666; *p* = 0.005
	*MIR127*	chr14:101349856	*rho* = 0.684; *p* = 0.042

*p,* adjusted level of significance (FDR-corrected). The inverse correlations are highlighted in gray. Strong correlations (|*rho*| > 0.7) are shown in *italics*. CpG sites with consistent correlations across two plaque instability indicators are shown in bold.

Within the *MIR127* region, a strong correlation (|*rho*| = 0.7.0.9) was observed only between methylation of chr14:101349561 and the number of neutrophils per field of view (Table 3). No consistent methylation changes associated with any instability parameter were detected across the entire *MIR127* region or across consecutive CpG sites. Nevertheless, three CpG sites demonstrated concordant associations with two independent markers of plaque instability. Methylation of chr14:101349509 negatively correlated with both fibrous cap thickness and necrotic core size, whereas methylation of chr14:101349643 negatively correlated with the numbers of macrophages and lymphocytes. Conversely, methylation of chr14:101349784 showed positive correlations with both macrophage and lymphocyte numbers (Table 3).

We next compared DNA methylation levels in carotid ASP from patients with and without a history of acute cerebrovascular events (ACVE). Significant differences were detected for seven individual CpG sites in the *MIR127* region (*p* < 0.046) (Figure 6A, orange boxes), whereas median methylation across the entire region did not differ between groups (*p* = 0.364) (Figure 6B). The observed methylation differences ranged from 2% to 4% and reached approximately 7% only for two adjacent CpG sites, chr14:101349509 (ACVE, 89.8 [85.0; 95.0]%, without ACVE, 82.1 [77.0; 89.2]%, *p* = 0.001) and chr14:101349515 (ACVE, 94.8 [91.3; 96.5]%, without ACVE 87.9 [84.1; 91.7]%, *p* = 0.046) (Figure 6A, red box). Notably, these same CpG sites also showed negative correlations with histological indicators of plaque instability. Methylation at both sites was inversely associated with fibrous cap thickness, whereas methylation at chr14:101,349,509 was additionally inversely associated with necrotic core size (Table 3).

**FIGURE 6:**
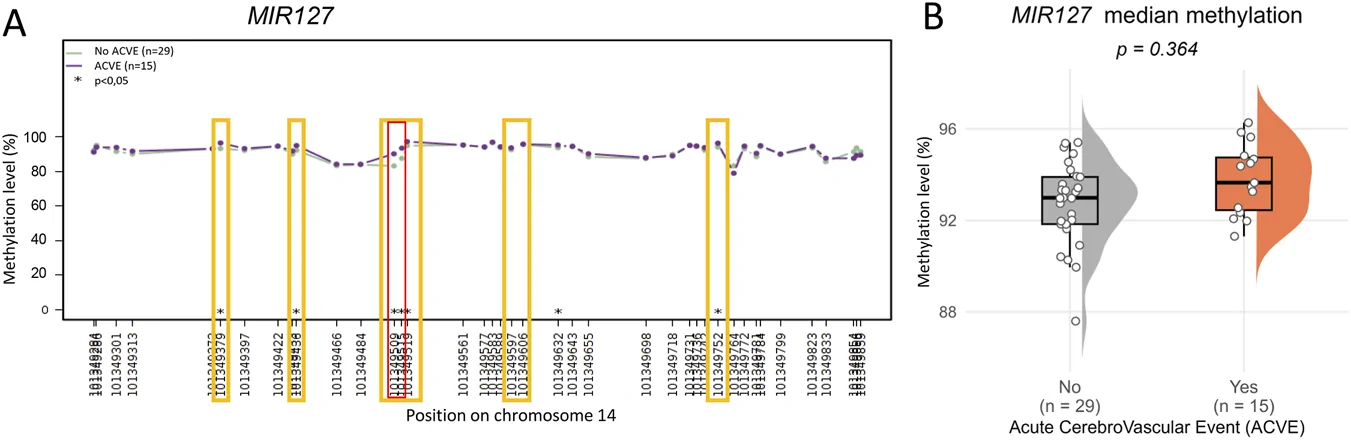
DNA methylation levels of *MIR127* gene in ASPs depending on the history of an acute cerebrovascular event (ACVE). **(A)** Methylation profile of CpG sites (The plot created in the online *Methylation plotter* tool (Mallona et al., 2014). **(B)** Median DNA methylation levels for the entire *MIR127* gene region in ASP.

At first glance, these findings appear contradictory, as increased methylation at chr14:101349509 was associated both with a history of ACVE and with reduced fibrous cap thickness, a feature of plaque instability, while also correlating with a smaller necrotic core, whereas unstable plaques are generally characterized by large lipid core (Boswell-Patterson et al., 2023). A possible explanation is that plaque vulnerability is commonly evaluated using the relative area occupied by the necrotic core (>25% of the plaque cross-sectional area) (Lovett et al., 2004; Gerhardt et al., 2022; Noonan et al., 2025), whereas in our study the necrotic core was measured as its absolute thickness (μm) on histological sections. These parameters may therefore reflect different morphological aspects of plaque composition and are not necessarily directly comparable.

#### 3.2.4 Increased DNA methylation of a single CpG site in *MIR100* E-box region in atherosclerotic plaque is associated with metabolic syndrome

The *MIR100* E-box region, a putative regulatory region of the *MIR100* gene, does not contain CpG sites annotated on Illumina methylation microarrays. Consequently, data regarding DNA methylation in this regulatory region remain limited.

Using BSAS, we assessed methylation at seven of the eight CpG sites in the *MIR100* E-box region, chr11:122025042 was excluded because of insufficient coverage. Among the analyzed sites, only chr11:122025143 demonstrated a significant association with clinical characteristics. Specifically, patients with metabolic syndrome exhibited higher methylation levels at this CpG site than patients without metabolic syndrome (92.2 [91.5; 92.6]% vs. 89.1 [86.7; 90.9]%, *p* = 0.021) (Figure 7). No significant associations were identified between methylation at this or any other CpG site in the region and the remaining clinical characteristics examined.

**FIGURE 7:**
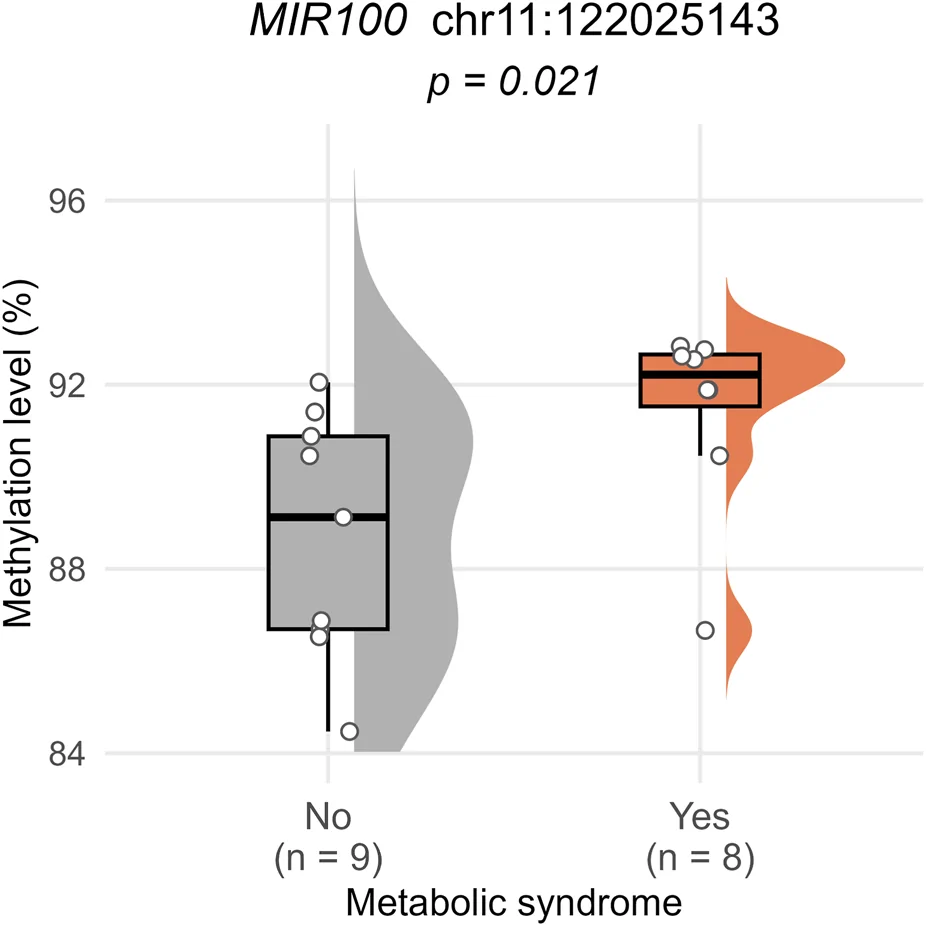
The methylation level of chr11:122025143 CpG site in the *MIR100* E-box region in the carotid artery ASPs depending on the presence of metabolic syndrome.

#### 3.2.5 Methylation of *MIR10B* and *MIR143HG (CARMN)* is correlated with expression of corresponding microRNAs

Lab-internal EWAS datasets comprised paired DNA methylation and microRNA expression data; however, due to inadequate RNA quality, microRNA expression was successfully assessed in 14 samples.

The most highly expressed microRNAs in all samples were miR-143–3p (18.6 [18.2; 19.5]; Q2 [Q1; Q3]), miR-21–5p (17.1 [16.5; 17.7]), and miR-100–5p (16.0 [15.5; 16.2]; Figure 8). Along with this primary triad, three additional microRNAs (miR-145–5p, miR-145–3p, and miR-10b-5p) also demonstrated robust accumulation, with median values successfully clustering above the 10 log2СPM baseline. In contrast, miR-143–5p, miR-127–3p, miR-21–3p, miR-127–5p, and miR-10b-3p fell below this threshold, displaying substantially lower expression levels.

**FIGURE 8:**
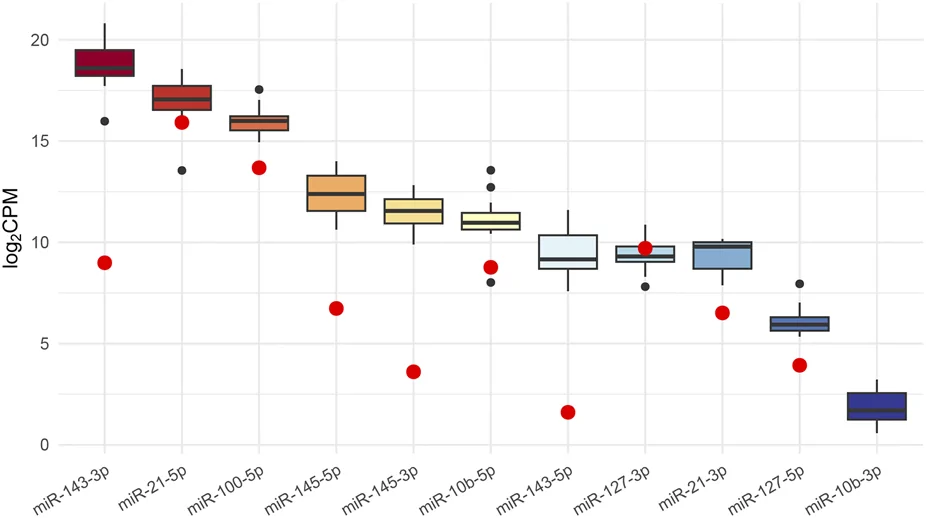
Expression levels of the top highly expressed microRNAs in carotid atherosclerotic plaques (n = 14). The boxplots represent log_2_CPM values, ordered by descending median expression. Red dots highlight altered microRNA expression levels during carotid artery restenosis.

Notably, a single plaque sample obtained from a patient with recurrent carotid artery restenosis (indicated by the red dots in Figure 8) exhibited a profound, coordinated downregulation across nearly all analyzed microRNAs. This drop was most pronounced for the smooth muscle cell-specific markers miR-143–3p, miR-145–5p, miR-145–3p, and miR-143–5p, as well as for miR-21–3p, and miR-127–5p.

Eight significant inverse correlations were identified between methylation of seven CpG sites and expression of four microRNAs: miR-10b-3p, miR-10b-5p, miR-143–3p, and miR-145–3p (Table 4).

**TABLE 4:** Significant correlations between the methylation of CpG sites of microRNA genes annotated on the Infinium HumanMethylation450 BeadChip and microRNA expression.

Location in the gene	CpG site (illumina Id)	microRNA	Spearman’s *rho*	*p* (FDR)
*MIR10B* promoter^#^	cg27160395	*miR-10b-3p*	−0.656	0.011
		*miR-10b-5p*	−0.653	0.014
*MIR143HG* (*CARMN*) promoter^#^	cg01726320	*miR-143-3p*	−0.569	0.037
		*miR-145-3p*	−0.705	0.006
	cg01558401	*miR-143-3p*	−0.622	0.020
		*miR-145-3p*	−0.749	0.003
*MIR143HG* (*CARMN*) 1st exon	cg26585864	*miR-143-3p*	−0.622	0.020
		*miR-145-3p*	−0.749	0.003
*MIR143HG* (*CARMN*) 3rd intron, *MIR143* promoter^#^	cg04317047	*miR-145-3p*	0.679	0.010
*MIR143HG* (*CARMN*) 4th exon, *MIR143* promoter^#^	cg16684117	*miR-143-3p*	0.569	0.037
		*miR-145-3p*	0.692	0.008
	cg21242144	*miR-143-3p*	0.631	0.018
		*miR-145-3p*	0.811	<0.001

# promoter regions were defined as 3 Kb upstream and 500bp downstream of miRNA, transcription start sites (Marsico et al., 2013; Huan et al., 2020).

For *MIR10B*, methylation of the promoter-associated cg27160395 site was inversely correlated with both miR-10b-5p and miR-10b-3p expression, consistent with previous reports linking cg27160395 hypermethylation to reduced miR-10 b expression (Kim et al., 2011; Li et al., 2015). Thus, reduced *MIR10B* promoter methylation in atherosclerotic plaques may be linked to increased miR-10 b expression.

Both positive and negative associations were observed between the methylation of *MIR143HG* (*CARMN*) and the expression of miR-143–3p and miR-145–3p. CpG sites near the transcription start site (TSS) of *MIR143HG* (cg01726320, cg01558401, and cg26585864) were inversely associated with the expression of both microRNAs, whereas sites within the *MIR143HG* gene body (cg04317047, cg16684117, and cg21242144) showed positive associations.

### 3.3 Methylation of microRNA genes in peripheral blood leukocytes in atherosclerosis

#### 3.3.1 Regions of *MIR10B*, *MIR21* putative promoter, *MIR21* CpG island and *MIR100* E-box hypomethylated in blood leukocytes of the patients with carotid atherosclerosis

The BSAS results revealed hypomethylation of individual CpG sites in the *MIR10B* region, *MIR21* CpG island and *MIR100* E-box region in PBP vs. PBC (Table 5). When examining the entire regions, a significant difference in median DNA methylation was observed only for the *MIR21* putative promoter (Table 5; Figure 2).

**TABLE 5:** Statistically significant hypomethylation in CpG sites and entire *MIR21* putative promoter region in peripheral blood leukocytes of the patients with advanced carotid atherosclerosis (PBP) compared with subclinical controls (PBC).

Region	CpG site	β in PBP	β in PBC	Δβ (*p* (FDR))
*MIR10B*	​	(n = 73)	(n = 27)	​
	cg12215739 (chr2:177014966)	10.2 [7.7; 16.4]%	17.0 [10.7; 22.8]%	−6.8 (*p* = 0.016)
	chr2:177015172	24.3 [19.0; 30.4]%	27.7 [25.2; 31.5]%	−3.4 (*p* = 0.012)
	chr2:177015208	19.1 [15.0; 27.9]%	29.4 [17.1; 33.3]%	−10.3 (*p* = 0.047)
*MIR21* putative promoter	​	(n = 81)	(n = 30)	​
	Median	30.3 [21.8; 39.3]%	36.8 [28.8; 46.0]%	−6.5 (*p* = 0.014)
	chr17:57915172	0.7 [0.1; 1.8]%	2.1 [0.6; 4.8]%	−1.4 (*p* = 0.005)
	chr17:57915371	3.6 [1.8; 4.5]%	6.9 [3.9; 11.2]%	−3.3 (*p* = 0.012)
	cg12054453 (chr17:57915717)	18.4 [10.5; 27.7]%	25.9 [23.6; 32.4]%	−7.5 (*p* = 0.015)
*MIR21* CpG island	​	(n = 35)	(n = 19)	​
	chr17:57916241	2.1 [1.2; 3.3]%	4.2 [3.5; 6.8]%	−2.1 (*p* = 0.001)
	chr17:57916306	3.6 [2.0; 7.3]%	8.7 [6.7; 12.7]%	−5.1 (*p* = 0.001)
	chr17:57916347	20.8 [16.8; 27.3]%	29.6 [26.7; 33.2]%	−8.8 (*p* < 0.001)
	chr17:57916385	49.1 [40.0; 56.1]%	55.6 [52.0; 60.7]%	−6.5 (*p* = 0.025)
	chr17:57916391	42.2 [30.7; 47.7]%	45.8 [43.5; 49.8]%	−3.6 (*p* = 0.036)
	chr17:57916416	63.3 [48.3; 68.2]%	67.7 [64.5; 72.9]%	−4.4 (*p* = 0.038)
	chr17:57916418	61.5 [47.6; 66.4]%	66.7 [59.8; 72.5]%	−5.2 (*p* = 0.030)
*MIR100* E-box	​	(n = 18)	(n = 17)	​
	chr11:122025072	95.9 [95.4; 97.2]%	97.1 [96.6; 98.0]%	−1.2 (*p* = 0.041)

β, methylation level; Δβ, differences in methylation level between groups, *p* (FDR), FDR-adjusted *p*-values.

We next compared our BSAS data with the whole-blood EWAS dataset GSE69138 comprising 404 patients with ischemic stroke, distributed across 3 stroke subtypes: cardioembolic (n = 127), lacunar (n = 141), and stroke caused by large artery atherosclerosis (n = 132) (Soriano-Tárraga et al., 2018).

Overall, the methylation patterns of the *MIR21* putative promoter region and *MIR143* regions were comparable between BSAS and EWAS (Figure 9). The largest discrepancies were observed for *MIR127* region, cg14399060 in *MIR10B* region and cg02515217 in the *MIR21* gene body. Compared with EWAS, BSAS showed lower methylation in the *MIR10B* region and *MIR21* gene body but higher methylation in *MIR127* region. These differences may partly reflect differences in disease stage, since samples in the current study were collected outside the acute cerebrovascular event (ACVE) period, whereas the external EWAS included patients sampled during the acute phase of stroke (Soriano-Tárraga et al., 2018).

A history of ACVE had little influence on leukocyte DNA methylation in our BSAS results. Significant differences were detected only for two CpG sites, both with small effect sizes (2.0%–3.1%): cg01152019 (chr2:177015044) in *MIR10B* region showed lower methylation (8.1 [6.8; 11.0]% vs. 11.2 [8.3; 13.8]%, p = 0.044), whereas chr14:101349436 in *MIR127* region showed higher methylation in patients with previous ACVE (96.4 [95.0; 97.4]% vs. 94.4 [92.2; 96.5]%, *p* = 0.033). No significant associations were observed for the remaining analyzed regions.

#### 3.3.2 DNA methylation level of the *MIR10B* and *MIR143* genes in blood leukocytes of patients with atherosclerosis is associated with blood lipids and degree of carotid stenosis

Next, we analyzed the associations between the DNA methylation level in the studied regions of microRNA in the in leukocytes of patients with atherosclerosis and pathogenetically relevant traits of atherosclerosis.

The analysis revealed strong negative correlations (*rho* < −0.7) for chr2:177015208 in the *MIR10B* gene with serum glucose, total cholesterol and atherogenic index (Table 6). For cg12215739 (chr2:177014966) we observed strong negative correlation with atherogenic index and moderate negative correlations (*rho* = −0.4-0.69) with total cholesterol level. Both cg12215739 (chr2:177014966) and chr2:177015208 showed moderate negative correlations with low-density lipoprotein levels and with the degree of carotid artery stenosis. There were also single correlations of methylation of other CpG sites in *MIR10B* gene region with stenosis degree or total cholesterol levels (Table 6).

**TABLE 6:** Correlations between DNA methylation of individual CpG sites in peripheral blood leukocytes of patients with carotid atherosclerosis and clinically relevant traits.

Trait	Region	CpG site	Spearman’s *rho* and *p* (FDR)
Age, years	*MIR127*	chr14:101349655	*rho =* −0.465; *p* = 0.008
	*MIR143* gene body	**chr5:148808456**	*rho =* −0.607; *p =* 0.047
		**chr5:148808486**	*rho =* −0.601; *p =* 0.039
Stenosis of carotid artery, %	*MIR10B*	**cg12215739 (chr2:177014966)**	*rho =* −0.490; *p =* 0.002
		**chr2:177015208**	*rho =* −0.470; *p <* 0.001
		chr2:177015141	*rho =* 0.506; *p <* 0.001
		chr2:177015213	*rho =* 0.466; *p <* 0.001
	*MIR127*	chr14:101349731	*rho* = 0.465; *p* = 0.001
	*MIR143HG* promoter	**chr5:148786518**	*rho= −0.764; p = 0.046*
		chr5:148786669	*rho= −0.761; p = 0.028*
	*MIR143* gene body	**chr5:148808474**	*rho = 0.726; p = 0.027*
Glucose, mmol/L	*MIR10B*	chr2:177015208	*rho= −0.701; p < 0.001*
Total cholesterol (TC), mmol/L	*MIR10B*	chr2:177014962	*rho =* −0.525; *p =* 0.006
		**cg12215739** **(chr2:177014966)**	*rho =* −0.627; *p <* 0.001
		**chr2:177015208**	*rho= −0.701; p < 0.001*
	*MIR143* gene body	**chr5:148808456**	*rho = 0.881; p = 0.007*
		**chr5:148808474**	*rho =* 0.678; *p =* 0.045
		**chr5:148808486**	*rho = 0.711; p = 0.031*
Low density lipoproteins (LDL), mmol/L	*MIR10B*	**cg12215739 (chr2:177014966)**	*rho =* −0.636; *p =* 0.026
		**chr2:177015208**	*rho =* −0.643; *p =* 0.024
	*MIR143* gene body	**chr5:148808456**	*rho = 0.786; p = 0.048*
Triglycerides, mmol/L	*MIR143HG* promoter	**chr5:148786518**	*rho= −0.886; p = 0.019*
Atherogenic index AI = $\frac{TC-HDL}{HDL}$	*MIR10B*	**cg12215739 (chr2:177014966)**	*rho= −0.772; p = 0.003*
		**chr2:177015208**	*rho= −0.789; p = 0.002*

HDL, high density lipoproteins, *p* (FDR), FDR-adjusted *p*-values. The inverse correlations are highlighted in gray. Sites associated with two and more features are shown in bold.

The strong negative correlations with degree of carotid artery stenosis and triglycerides levels were revealed for methylation level of 2 CpG sites in *MIR143HG* promoter, and the chr5:148786518 showed strong correlation with both traits. At the same time, the CpG sites of *MIR143* gene body showed positive correlations with stenosis degree and parameters of lipid metabolism (total cholesterol and low-density lipoproteins). The moderate negative correlations with patients age were also noted for the CpG sites of *MIR143* gene body (Table 6).

None of CpG sites in the *MIR127* gene region showed significant correlation with more than one examined clinical trait (Table 6). In addition, no DNA methylation correlations observed in leukocytes of patients with advanced atherosclerosis were replicated in leukocytes of the subclinical controls (all *p* > 0.084).

#### 3.3.3 *MIR21* gene body and *MIR143* gene body methylation levels are associated with cellular composition of blood

Peripheral blood is a frequently used material for analysis, but variation in leukocyte composition may influence whole-blood DNA methylation. To assess this potential source of variation, cell-type proportions were estimated in the external EWAS dataset GSE246337 using a DNAm reference matrix designed for adult whole blood implemented in the EpiDISH algorithm, which estimates fractions of seven immune cell types (B cells, NK cells, CD4^+^ and CD8^+^ T cells, monocytes, neutrophils, and eosinophils) (Teschendorff et al., 2017). The blood cell estimation of external EWAS (GSE246337) was evaluated by 28 CpG sites of microRNA genes annotated on Illumina methylation microarrays (Supplementary Table S14).

Methylation at two CpG sites in the *MIR143* gene body, cg16684117 and cg21242144, showed strong positive correlations with the estimated proportions of B cells and CD4T cells (Figure 10). In vascular tissue, the same CpG sites showed strong positive correlations with the estimated SMC proportion (Figure 4). Methylation at cg01558401 in the *MIR143HG* promoter was also associated with the estimated proportion of peripheral blood neutrophils and IC infiltration in carotid plaques (Figures 4, 10), suggesting that methylation variation at these sites may partly reflect cellular composition.

**FIGURE 9:**
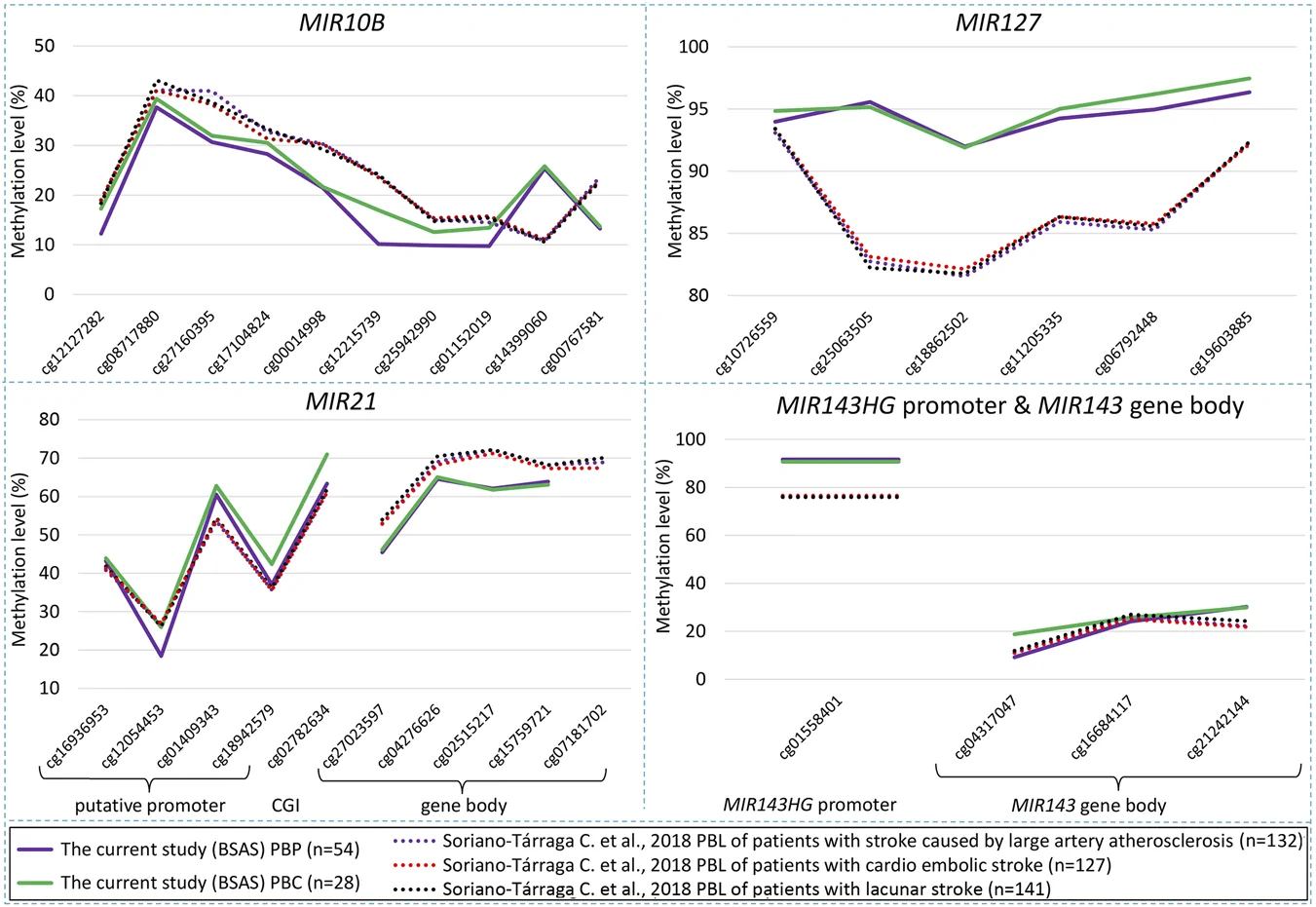
Comparison of DNA methylation at CpG sites annotated on Illumina methylation microarrays in the *MIR10B*, *MIR21*, *MIR127*, and *MIR143* regions in peripheral blood leukocytes in the current BSAS study and external EWAS dataset. BSAS, bisulfite amplicon sequencing; PBP, peripheral blood leukocytes of patients; PBC, peripheral blood leukocytes of subclinical controls. Note: Axis ranges are adjusted for each region to improve visualization of within-region differences; therefore, absolute methylation levels should not be compared visually across panels.

**FIGURE 10:**
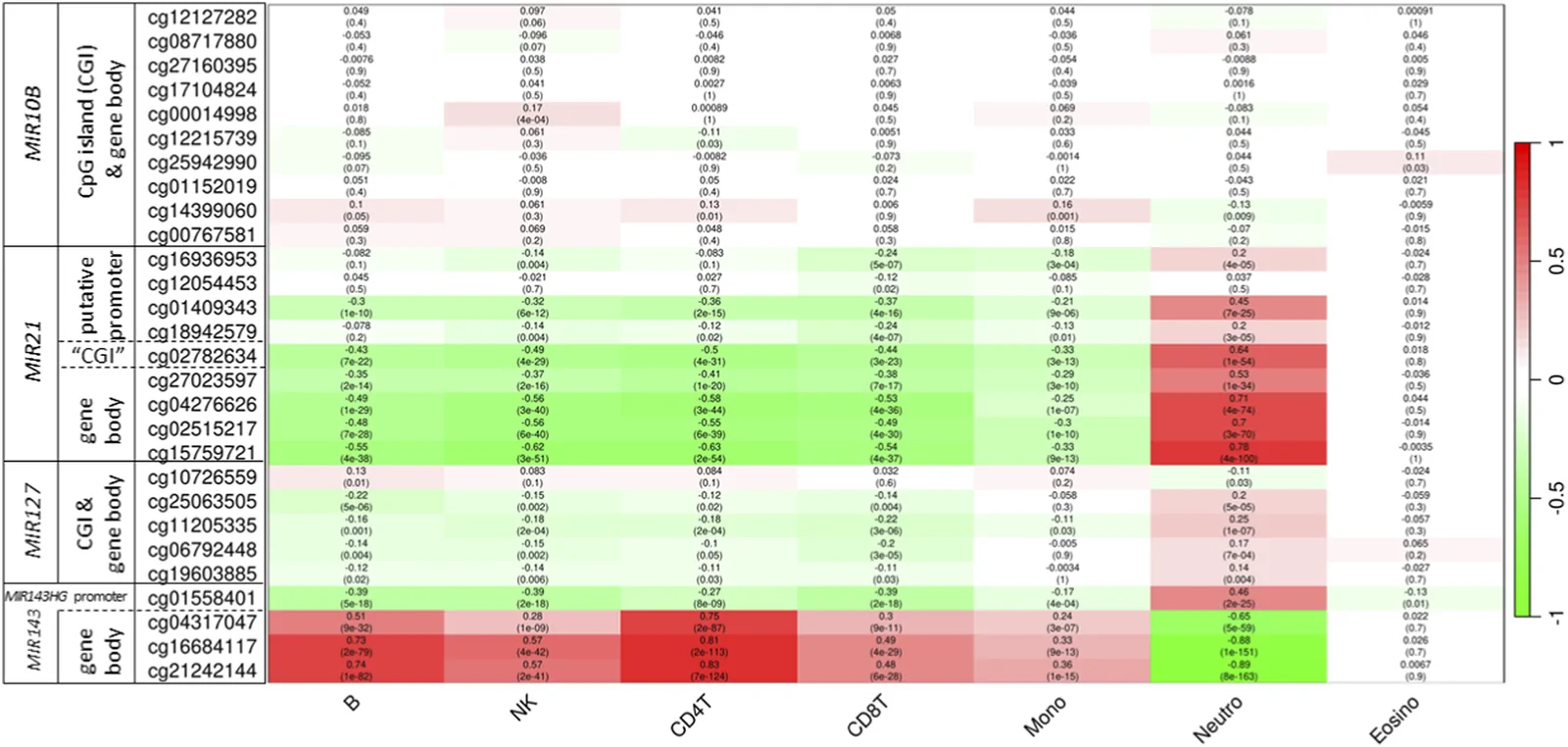
Heatmap of correlations between DNA methylation of analyzed CpG sites and the main blood cell types in peripheral blood–B cells, NK, CD4T cells, CD8T cells, monocytes (Mono), neutrophils (Neutro), eosinophils (Eosino).

These findings indicate that cellular composition is an important potential source of variation in methylation at some miRNA-associated CpG sites in peripheral blood.

#### 3.3.4 *MIR21* CpG island methylation in peripheral blood reflects its methylation status in carotid atherosclerotic plaques

Using our BSAS results, we compared the methylation of the studied microRNA regions in atherosclerotic plaques of patients and in their peripheral blood (ASP vs. PBP). Significant positive correlations were observed for the median methylation levels of *MIR10B* region (*rho* = 0.336, *p* = 0.009), *MIR21* putative promoter *(rho* = 0.432, *p* < 0.001), *MIR21* CpG island (*rho* = 0.722, *p* < 0.001) and *MIR143* gene body (*rho* = 0.482, *p* = 0.038) (Supplementary Table S01).

The strongest correlations (*rho* > 0.7) were observed for cg14399060 in *MIR10B* gene (chr2:177015070, *rho* = 0.841, *p* < 0.001) and four consecutive CpG sites in the *MIR21* CpG island: chr17:57916598 (*rho* = 0.745, *p* < 0.001), chr17:57916641 (*rho* = 0.745, *p* < 0.001), cg02782634 (chr17:57916643, *rho* = 0.702, *p* < 0.001) and chr17:57916647 (*rho* = 0.731, *p* < 0.001). Notably, cg14399060 was also associated with thrombus on the plaque surface (Table 2) and differed in peripheral blood leukocytes between our BSAS data and the external stroke EWAS dataset (Soriano-Tárraga et al., 2018).

These findings suggest that peripheral blood leukocytes, as an easily accessible source for DNA analysis, may serve as a surrogate material for indirectly assessing DNA methylation in carotid plaques. Four CpG sites and the entire *MIR21* CpG island region seem to be the most suitable for this purpose.

## 4 Discussion

DNA methylation of microRNA genes has been investigated predominantly in cancer, whereas studies addressing vascular tissues and peripheral blood leukocytes in atherosclerosis remain scarce. Consequently, the epigenetic regulation of microRNA genes in atherosclerosis is still poorly understood. To our knowledge, this study represents one of the most comprehensive targeted analyses of DNA methylation across multiple microRNA gene regions in advanced carotid atherosclerosis. A major strength of the present study is the simultaneous analysis of vascular tissues and peripheral blood leukocytes obtained from the same patients with carotid atherosclerosis. In addition to microRNA gene bodies, we investigated their regulatory elements, including CpG islands, promoter regions, and a transcription factor binding site. Furthermore, DNA methylation profiles across these targeted loci of five microRNA genes (*MIR10B, MIR21, MIR100, MIR127,* and *MIR143*) were integrated with paired microRNA expression data within the atherosclerotic plaques, enabling a focused assessment of the epigenetic mechanisms regulating the transcription of these specific microRNAs within the vascular wall.

Our results demonstrate that advanced carotid atherosclerosis is characterized by a moderate DNA hypomethylation within the vascular wall, affecting the *MIR10B* and *MIR127* regulatory regions, the *MIR100* E-box element, and the gene bodies of *MIR21* and *MIR143*. In peripheral blood, similarly moderate DNA methylation changes were observed, featuring hypomethylation of individual CpG sites within the *MIR10B* region, *MIR21* putative promoter, *MIR21* CpG island, and *MIR100* E-box region, although regional differences were significant only for the *MIR21* putative promoter. Thus, while DNA methylation changes were observed in both vascular tissue and blood, their specific magnitude and regional distribution differed between tissues.

Importantly, accounting for estimated cell composition refined the nature of these associations, separating cell-type abundance from cell-functional states. Specifically, *MIR100* E-box region and *MIR127* gene methylation remained associated with carotid atherosclerosis after adjustment for estimated vascular cell composition. These findings provide further support in our study that methylation at these sites is associated with atherosclerosis independently of the estimated vascular cell composition. Nevertheless, because cell proportions were computationally estimated, residual confounding by uncharacterized cellular subsets and other unmeasured factors cannot be completely excluded.

The deconvolution analysis of vascular tissue revealed a striking mirror-image dichotomy, where higher DNA methylation in the *MIR10B* and *MIR127* (except cg25063505) loci and the *MIR143* gene body serves as an epigenetic signature of SMCs, whereas methylation of the *MIR21* and *MIR143HG* promoters reflect IC infiltration. The opposite correlation of the DNA methylation of the *MIR143HG* promoter/*MIR143* gene body with ICs/SMCs likely reflects the cell-specific epigenetic regulation of gene. This finding aligns with the hypothesis that DNA methylation within microRNA gene bodies can actively foster transcription (Huan et al., 2020).

Peripheral blood DNA methylation profiles of the most analyzed microRNA genes are profoundly shaped by leukocyte composition. While the methylation variations within the *MIR21*, *MIR127* and *MIR143* loci are strongly confounded by specific IC fractions, mainly neutrophils, the *MIR10B* region remains robust and unaffected by cellular shifts. These findings emphasize the importance of accounting for cellular heterogeneity in epigenetic studies of both vascular and blood tissues.

Among the investigated genes, *MIR127* has been the most extensively studied in atherosclerosis. Previous analyses of femoral atherosclerotic plaques demonstrated hypomethylation of the 14q32 locus containing *MIR127* (Aavik et al., 2014). However, only relative fold changes rather than absolute methylation levels were reported, limiting direct comparison with our results. Another study described relatively low (<60%) methylation across several regions within the 14q32 locus in different vascular tissues: lower limb arteries and veins, upper limb veins and mammary arteries (Goossens et al., 2019). However, carotid arteries were not examined, and the regions analyzed differed from the CpG island investigated in the present study: the closest regions were located approximately 57 kb upstream and 22 kb downstream of the *MIR127* gene body and therefore did not overlap the region analyzed here. In contrast, we observed consistently high methylation (>80%) across nearly all CpG sites within the *MIR127* region in all studied tissues, including affected and intact carotid arteries. Notably, publicly available microarray datasets (GSE66500, GSE149759, GSE69138) demonstrated similarly high methylation levels for Illumina-annotated CpG sites within this region (Figures 3, 8), supporting the view that high constitutive methylation is a characteristic feature of the *MIR127* region despite minor differences between studies and analytical platforms.

Published data on DNA methylation of the *MIR100* regulatory region are extremely limited. Chen et al. (2014) reported hypermethylation of the *MIR100HG* (miR-100 host gene) in breast cancer (Chen et al., 2014). However, the genomic coordinates of the affected region were not specified, preventing direct comparison with our data. In the present study, we identified a low-magnitude increase in methylation at a single CpG site (chr11:122025143) in the *MIR100* E-box region in plaques from patients with metabolic syndrome. Because this E-box is bound by the transcription factor ZEB1, which has been reported to enhance miR-100 expression (Chen et al., 2014), altered methylation at this site could potentially affect regulatory activity. Reduced miR-100 expression has previously been reported in macrophage- and lipid-rich atherosclerotic plaques and in metabolic conditions associated with obesity and diabetes (Pankratz et al., 2018; Pek et al., 2016). Experimental studies have also suggested a protective role of miR-100 against high-fat diet-induced metabolic syndrome (Smolka et al., 2021). These observations provide a potential biological context for the association observed in our study.

For the *MIR10B* region, we observed a significant decrease in the DNA methylation level in atherosclerosis-affected carotid arteries. This finding is consistent with our previous study, in which methylation of four CpG sites within the *MIR10B* gene body and CpG island was lower in coronary atherosclerotic plaques than in other vascular tissues (Nazarenko et al., 2015). These observations suggest that reduced methylation of the *MIR10B* locus may represent a common epigenetic feature of advanced atherosclerosis across different vascular beds.

In the present study, methylation of a promoter-associated *MIR10B* CpG site was inversely correlated with miR-10b-3p and miR-10b-5p expression in advanced carotid atherosclerotic plaques. To explore the potential biological relevance of this association, we searched for the experimentally validated targets of miR-10b-5p using miRDB (mirdb.org), TarBase v.9.0 (dianalab.e-ce.*u*th.gr/tarbasev9/interactions), and miRTarBase v.9.0 (mirtarbase.cuhk.edu.cn). Four targets with strong supporting evidence were identified: *HOXD10, KLF4, NCOR2,* and *E2F7*. Given the reported involvement of miR-10 b in endothelial cell migration and angiogenesis, smooth muscle cell proliferation, and cholesterol metabolism (Shen et al., 2011; Wang et al., 2012; Yu et al., 2015; Nakahara et al., 2018), these findings provide a potential biological context for the observed methylation-expression association and its possible relevance to atherosclerosis.

The contrasting associations within the *MIR143HG (CARMN)* locus reveal a spatially coordinated mechanism of epigenetic regulation, where the methylation of CpG sites near the TSS correlates inversely with miR-143–3p and miR-145–3p expression, while gene-body methylation shows a strong positive correlation. Crucially, this locus-specific dual pattern is a novel finding of the present study. This pattern is partly consistent with previous evidence of a positive association between methylation of CpGs located within microRNA gene bodies and expression of these microRNAs in whole blood from the Framingham Heart Study (Huan et al., 2020). However, in the present study, this pattern appears to be specific to the host gene *MIR143HG*, with no clear relationship between CpG site location and microRNA expression observed for *MIR143* gene.

Data on methylation of the *MIR143HG* promoter and the *MIR143* gene body remain limited. One study analyzed DNA methylation of *MIR145* and the *MIR143HG* promoter by pyrosequencing in smooth muscle cells from saphenous veins of patients with type 2 diabetes mellitus but could not obtain reliable measurements for the *MIR143HG* promoter (Riches et al., 2017). Technical limitations also prevented assessment of *MIR145* methylation in the current study. Previous genome-wide analyses identified several hypermethylated regions within the long non-coding RNA *CARMN* (*MIR143HG*), which overlaps the sequences encoding miR-145 and miR-143, although the exact genomic coordinates were not reported, precluding direct comparison (Ehrlich et al., 2019). Our study successfully bridges this gap by providing high-resolution, position-specific methylation data linked directly to mature microRNA expression.

To explore the potential downstream consequences of this epigenetic disruption, we performed an *in silico* target screening. Among miRDB, TarBase v.9.0, and miRTarBase v.9.0, only vasohibin 1 (*VASH1*) was identified as a validated target of miR-143–3p. It inhibits the migration, proliferation, and network formation of vascular endothelial cells. Increased *VASH1* expression in arterial tissues and endothelial cells of carotid atherosclerotic plaques has been associated with neovascularization, inflammation, and plaque instability (Fukumitsu et al., 2015).

Information regarding *MIR21* methylation (both in the putative promoter region, CpG island and the gene body) in vascular tissues or peripheral blood leukocytes from patients with atherosclerosis has remained limited. Several of the analyzed CpG sites have previously been implicated in disease- or phenotype-associated methylation studies. In particular, cg12054453 and cg18942579, both located within the *VMP1/MIR21* region, have been associated with inflammatory bowel disease and lipid-related phenotypes in independent studies (Ventham et al., 2016; Wu et al., 2022). Other CpG sites analyzed in the present study are located within putative regulatory or miRNA-associated genomic regions, but their specific functional consequences have not been experimentally established.

Beyond differences in methylation between vascular tissues, we identified significant associations between DNA methylation and clinically relevant characteristics of plaque instability. Hypomethylation of individual CpG sites within *MIR10B* and the *MIR21* putative promoter was associated with thrombus formation on the plaque surface, intraplaque hemorrhage, and the presence of foam cells. In addition, two CpG sites in the *MIR127* region (chr14:101349509 and chr14:101349515) exhibited increased methylation in patients with a history of ACVE and were also associated with histological indicators of plaque instability. These significant associations underscore the high translational potential of microRNA gene methylation profiles, providing a strong foundation for future longitudinal studies to establish their clinical utility as predictive biomarkers for plaque destabilization.

Only one previous study has investigated methylation of microRNA genes in peripheral blood leukocytes from patients with cardiovascular disease. That genome-wide study using the Human Methylation450 BeadChip microarray identified a nominal association for cg16936953 (chr17:57915665) within the *MIR21* putative promoter in myocardial infarction (*p* = 0.049); however, significance was lost after correction for multiple testing (Ek et al., 2016). As in our study, methylation levels were lower in individuals with the disease. Ek et al. (2016) reported that cg16936953 is located in active promoter in ENCODE cell lines, supporting a potential regulatory role for this CpG in *MIR21*.

Peripheral blood leukocytes are exposed to systemic inflammatory and metabolic triggers, such as hyperlipidemia, which can induce epigenetic remodeling prior to or during atherogenesis. In our cohort, blood-derived methylation of selected CpG sites within *MIR10B*, the *MIR143HG* promoter, and the *MIR143* gene body was also associated with blood lipid parameters and the degree of carotid stenosis. While these associations should be interpreted as exploratory, particularly where individual CpG sites rather than entire functional regions were involved, they highlight the potential clinical utility of blood-based epigenetic biomarkers.

Overall, our BSAS and lab-internal EWAS data showed good agreement with external EWAS datasets regarding methylation levels and patterns of *MIR10B*, *MIR21*, *MIR127*, and *MIR143* in leukocytes (Soriano-Tárraga et al., 2018) and vascular tissues (Zaina et al., 2014; Zaina et al., 2015; Li et al., 2021). Some discrepancies between studies likely reflect methodological differences between bisulfite amplicon sequencing and microarray-based approaches, as well as biological differences among vascular beds and patient populations. Nevertheless, the overall concordance between datasets supports the reproducibility of the major methylation patterns observed in this study.

As thoroughly reviewed by Breton et al. (2017), small-magnitude effect sizes (1%–5% differences) in DNA methylation are highly characteristic of complex, non-malignant chronic diseases analyzed in peripheral blood. Such low-level shifts are functional and robustly validate gene-expression changes (Breton et al., 2017). Therefore, the relatively modest effect sizes observed at some CpG sites should not by themselves exclude biological relevance. In our study, the potential biological relevance of selected methylation changes was further supported by their associations with microRNA expression and by concordance with previously reported disease- or phenotype-associated methylation patterns.

An additional finding of potential translational interest was the concordance between DNA methylation in peripheral blood leukocytes and matched atherosclerotic plaques. Significant blood-plaque correlations were observed for several regions, with the strongest concordance for the *MIR21* CpG island. These findings suggest that selected CpG sites in this region may warrant further investigation as surrogate indicators of plaque methylation.

The study has several limitations that should be considered when interpreting the results. First, the relatively modest sample size reduced statistical power, particularly for subgroup analyses involving acute cerebrovascular events or specific histological features. Second, only a limited number of microRNA genes and regulatory regions were investigated, while technical limitations prevented analysis of the *MIR100* gene body and *MIR145* gene body. Broader epigenome-wide studies may therefore reveal additional DNA methylation changes relevant to carotid atherosclerosis. Third, the observational and cross-sectional design does not allow causal or temporal relationships to be established between DNA methylation, atherosclerosis, and clinical characteristics. In addition, the control group should not be regarded as completely atherosclerosis-free, as participants had subclinical carotid atherosclerosis. Accordingly, the blood-based comparisons reflect differences between advanced and early-stage carotid atherosclerosis and cannot establish DNA methylation changes associated with disease onset. Although vascular cellular heterogeneity was assessed using computational deconvolution and estimated cell proportions were incorporated into relevant regression models, these estimates are not direct measurements of cell abundance and residual confounding cannot be completely excluded. Direct cell-composition estimates were not available for the BSAS samples and therefore could not be incorporated as covariates in the primary BSAS analyses. The relatively limited number of paired blood and plaque samples also restricts the generalizability of the blood–plaque methylation correlations. Finally, functional experiments linking DNA methylation to downstream biological effects and independent experimental validation of the identified differentially methylated CpG sites were beyond the scope of the present study. Targeted validation of representative CpG sites using an independent DNA methylation assay in a larger independent cohort will be an important direction for future studies.

Several strengths of this study should be highlighted. First, the unique intra-patient paired design enabled simultaneous characterization of DNA methylation across atherosclerotic plaques, macroscopically intact adjacent carotid tissue, and peripheral blood leukocytes obtained from the same patients, allowing tissue-specific methylation patterns to be examined within a common clinical background. The availability of microRNA expression data from the same patients further enabled direct assessment of the relationship between DNA methylation and microRNA expression. Second, the targeted approach extended beyond conventional gene-body methylation analysis to encompass regulatory regions, including CpG islands, promoter-associated regions, and the E-box region of *MIR100*, providing a more comprehensive characterization of methylation across selected microRNA loci. Third, the integration of reference-based cell-type deconvolution allowed the potential contribution of cellular heterogeneity to methylation differences to be evaluated and helped identify CpG sites whose associations with carotid artery state persisted after adjustment for estimated vascular cell proportions. Finally, integration of the BSAS findings with independent publicly available EWAS datasets (GEO) provided cross-cohort and cross-platform comparison and identified patterns of concordance across different study populations and experimental approaches.

Taken together, this study provides a detailed characterization of DNA methylation patterns across selected microRNA gene regions in advanced carotid atherosclerosis and highlights the importance of tissue and cellular context when interpreting these patterns. The observed associations with plaque characteristics, microRNA expression, and blood-plaque concordance provide additional biological and translational context. Further studies in larger independent cohorts and with functional validation will be required to determine the biological significance and potential clinical applicability of these findings.

## 5 Conclusion

This study demonstrates tissue-specific moderate DNA methylation alterations featuring hypomethylation in microRNA regulatory regions associated with advanced carotid atherosclerosis. DNA methylation profiles of the analyzed regions of microRNA genes are profoundly shaped by cellular composition in both vascular tissues and blood. After adjustment for vascular cellular heterogeneity, methylation changes within the *MIR100* and the *MIR127* genes remained independently associated with carotid atherosclerosis. DNA methylation within the *MIR10B* exhibited weak correlation with blood cell composition.

In carotid atherosclerotic plaques methylation changes within *MIR100* were associated with metabolic syndrome, while alterations in *MIR10B*, *MIR21*, and *MIR127* were linked to histological plaque instability and history of acute cerebrovascular events. In blood, *MIR10B* and *MIR143* methylation were associated with lipid metabolism and carotid stenosis, whereas methylation of the *MIR21* CpG island showed strong correspondence between blood and plaque, supporting its potential as a surrogate biomarker in future studies.

Our findings provide evidence of an association between DNA methylation at *MIR10B* promoter and *MIR143HG (CARMN)* with the expression of their corresponding microRNAs, which represent the dominant, highly expressed profiles in vascular tissue. The downregulation of the contractile smooth muscle cell cluster (miR-143/145) observed in the pathological vascular tissue can be explained by an epigenetic switch mechanism, characterized by canonical silencing via promoter hypermethylation alongside alternative intronic/exonic gene-body regulation.

## Data Availability

The datasets presented in this article are not readily available because The datasets generated and analyzed during the current study are subject to restrictions due to applicable legal and ethical requirements governing the handling and transfer of human genetic data. Individual-level BSAS sequencing data and individual-level DNA methylation data generated using Illumina methylation arrays (including MethylationEPIC BeadChip microarray) cannot be deposited in publicly accessible repositories or transferred outside the Russian Federation. Therefore, the raw sequencing and individual-level methylation datasets are not publicly available. Detailed methodological information, genomic coordinates of the analyzed CpG sites, and summary-level results are provided in the article and Supplementary Material. The external datasets analyzed in this study are publicly available through the Gene Expression Omnibus (GEO) under accession numbers GSE46394, GSE66500, GSE149759, GSE69138, and GSE246337. Requests to access will be considered subject to applicable legal and ethical restrictions.
