# Supplementary material for: Tissue-specific moderate DNA methylation alterations featuring hypomethylation in microRNA regulatory regions associated with advanced carotid atherosclerosis
Source: Front Cell Dev Biol. 2026 Oct 19;14:1948449. doi: 10.3389/fcell.2026.1948449 (PMC13724618; doi:10.3389/fcell.2026.1948449)
Supplement: Supplementary file 1 [file DataSheet1.PDF]

Supplementary Material

Supplementary Methods

1 Study participants.

This study included 92 patients with advanced carotid atherosclerosis and 32 subclinical atherosclerosis controls (**Table SM1**). Participants were recruited at the Research Institute of Complex Problems of Cardiovascular Diseases (Kemerovo, Russia) and the Cardiology Research Institute (Tomsk National Research Medical Center, Tomsk, Russia) in 2014-2017. All participants provided written informed consent in accordance with the principles of the Declaration of Helsinki.

Exclusion criteria were non-Caucasian ethnicity or first-degree relationship with another study participant, age less than 40 years, presence of acute severe comorbid somatic pathology or exacerbation of a chronic disease; or refusal to participate.

Table SM1. Clinical characteristics of the study groups included

Parameter	Patients with advanced carotid atherosclerosis (n=92)	Subclinical atherosclerosis group (n=32)	*p* value
**Clinical parameters**			
Men: Women	64:28	19:13	0.402
Age, years (M [Q1; Q3])	64.0 [60.0; 69.0]	67.0 [58.8; 71.8]	0.196
Body mass index, kg/m^2 (M [Q1; Q3])	28.4 [25.9; 31.6]	26.2 [23.4; 29.1]	**0.012**
Metabolic syndrome [n (%)]	61 (66.3%)	*no data*	—
Waist circumference, cm (M [Q1;Q3])	101.0 [93.0; 113.0]	*no data*	—
History of angina [n (%)]	61 (66.3%)	0 (0.0%)	**0.002**
Angina functional class [n (%)]<br>- I FC<br>- II FC<br>- III FC	 12 (13.0%)<br>35 (38.0%)<br>14 (15.2%)	*no data*	—
History of myocardial infarction [n (%)]	36 (39.1%)	2 (6.3%)	**<0.001**
History of coronary artery disease [n (%)]	75 (81.5%)	5 (15.6%)	**<0.001**
Duration of coronary artery disease, years (M [Q1;Q3])	2.5 [1.0; 9.8]	*no data*	—
History of chronic heart failure [n (%)]	76 (82.6%)	1 (3.1%)	**<0.001**
Chronic heart failure functional class [n (%)]<br>- I FC<br>- II FC<br>- III FC	 6 (6.5%)<br>63 (68.5%)<br>8 (8.7%)	*no data*	—
History of TIA or stroke [n (%)]	26 (28.3%)	0 (0.0%)	**<0.001**
Hypertension [n (%)]	90 (97.8%)	15 (46.9%)	**<0.001**
Duration of hypertension, years (M [Q1; Q3])	13.5 [7.3; 20.0]	*no data*	—
Hypercholesterolemia [n (%)]	63 (68.5%)	5 (15.6%)	—
Smoking [n (%)]<br>including former smokers	60 (65.2%)<br>13 (14.1%)	11 (34.4%)<br>0 (0.0%)	**0.002**<br>0.020

Diabetes mellitus [n (%)]	22 (23.9%)	3 (9.4%)	0.123
Chronic limb ischemia [n (%)]	19 (20.7%)	*no data*	—
**Vascular ultrasound parameters (M [Q1; Q3])**			
Carotid artery stenosis, %	85 [80; 95]	28 [24; 45]	**<0.001**
Intima-media complex thickness, mm	*no data*	0.8 [0.7; 0.9]	—
**Laboratory data (M [Q1; Q3])**			
Glucose, mmol/l	5.8 [5.3; 7.3]	5.4 [5.1; 5.7]	**0.017**
Total cholesterol, mmol/L	5.3 [4.0; 6.3]	5.2 [4.7; 5.7]	0.898
Triglycerides, mmol/L	1.5 [1.3; 2.0]	1.3 [1.0; 1.5]	0.077
LDL, mmol/L	2.9 [2.0; 3.7]	3.1 [2.9; 3.7]	0.641
HDL, mmol/L	1.0 [1.0; 1.1]	1.4 [1.1; 1.6]	**0.017**
Atherogenic index (AI = (Total cholesterol - HDL)/HDL)	4.8 [2.4; 5.3]	2.7 [2.3; 3.3]	0.140
**Medications [n (%)]**			
Aspirin	65 (70.7%)	0 (0.0%)	**<0.001**
Clopidogrel	25 (27.2%)	1 (3.1%)	**<0.001**
Warfarin	10 (10.9%)	0 (0.0%)	0.059
Beta-blockers	57 (62.0%)	5 (15.6%)	**<0.001**
Angiotensin converting enzyme inhibitors	44 (47.8%)	2 (6.3%)	**<0.001**
Angiotensin II receptor blockers	27 (29.3%)	5 (15.6%)	0.160
Calcium channel blockers (dihydropyridine)	47 (51.1%)	1 (3.1%)	**<0.001**
Calcium channel blockers (non-dihydropyridine)	9 (9.8%)	0 (0.0%)	0.110
Digoxin	11 (12.0%)	0 (0.0%)	**0.035**
Loop diuretics	14 (15.2%)	0 (0.0%)	**0.020**
Thiazide diuretics	18 (19.6%)	2 (6.3%)	0.096
Spironolactone	14 (15.2%)	0 (0.0%)	**0.020**
Nitrates	9 (9.8%)	0 (0.0%)	0.110
Statins	67 (72.8%)	1 (3.1%)	**<0.001**
Cordarone	17 (18.5%)	0 (0.0%)	**0.006**
Insulin	9 (9.8%)	0 (0.0%)	0.110
Metformin	16 (17.4%)	1 (3.1%)	0.039

Abbreviations: n, number of individuals in the group; M, median; Q1, first quartile (25th percentile); Q3, third quartile (75th percentile).
FC, functional class; HDL, high density lipoproteins; LDL, low density lipoproteins; TIA, transient ischemic attack.

2 Histological examination of carotid atherosclerotic plaques

Histological examination was performed for 56 carotid ASP samples (60.7 %). Tissue fragments were fixed in 10% neutral formalin, processed in a Shandon Excelsior ES Tissue Processor (Thermo Scientific, USA), and embedded in paraffin. Serial 5-µm paraffin sections (5-6 microns) were prepared using a Microm HM 430 microtome (Thermo Scientific, USA), stained with hematoxylin-eosin and Van Gieson's stain, examined using an Axio Lab.A1 microscope (Carl Zeiss, Germany), and imaged with a MIRAX MIDI slide scanner (Carl Zeiss, Germany). Morphometric analysis was performed using ImageJ (NIH, USA). Plaques were classified according to the American Heart Association system (AHA) (Stary et al. 1995).

Table SM2. Morphological characteristics of carotid atherosclerotic plaques (N=16) included in the MethylationEPIC and miRNA-seq analysis

**Assessment of the atherosclerotic plaque cap**	
Cap thickness, microm (M [Q1;Q3])	308.5 [236.2; 468.6]
Thinnings [n (%)]	11 (68.8%)
Ruptures [n (%)]	8 (50.0%)
Erosions [n (%)]	6 (37.5%)
Ulcerations [n (%)]	5 (31.3%)
Calcifications [n (%)]	1 (6.3%)
**Assessment of the atherosclerotic plaque core**	
Core size, microm (M [Q1;Q3])	1803.5 [1087.9; 2234.8]
Calcifications [n (%)]	4 (25.0%)
**Assessment of the atherosclerotic plaque shoulder**	
Ruptures [n (%)]	7 (43.8%)
Thrombus [n (%)]	3 (18.8%)
Hemorrhages [n (%)]	7 (43.8%)
Calcifications [n (%)]	2 (12.5%)
**Assessment of inflammatory infiltration in atherosclerotic plaque**	
Leukocyte infiltration [n (%)]	16 (100.0%)
Degree of leukocyte infiltration [n (%)]:	
+	8 (50.0%)
++	8 (50.0%)
+++	0 (0.0%)
Plaques containing macrophages [n (%)]	12 (75.0%)
Number of macrophages per field of view (M [Q1;Q3])	3.0 [3.0; 4.0]
Plaques containing lymphocytes [n (%)]	16 (100.0%)
Number of lymphocytes per field of view (M [Q1;Q3])	9.5 [6.0; 12.3]
Plaques containing neutrophils [n (%)]	8 (50.0%)
Number of neutrophils per field of view (M [Q1;Q3])	2.0 [1.0; 2.0]
Plaques containing foam cells [n (%)]	5 (31.3%)
Number of foam cells per field of view (M [Q1;Q3])	5.0 [3.0; 10.0]
**Assessment of neovascularization sites**	
Plaques with neovascularization sites	6 (37.5%)
Recanalization [n (%)]	1 (6.3%)
Recanalization + endothelium [n (%)]	1 (6.3%)

Assessment of fibrosis	
The degree of the vessel fibrosis [n (%)]	
+	3 (18.8%)
++	11 (68.8%)
+++	2 (12.5%)
The degree of the lipid core fibrosis [n (%)]	
+	7 (43.8%)
++	7 (43.8%)
+++	2 (12.5%)
The degree of collagen fragmentation [n (%)]:	
+	5 (31.3%)
++	6 (37.5%)
+++	5 (31.3%)
AHA plaque type (Stary et al. 1995) [n (%)]:	
III,	1 (6.3%)
IV,	6 (37.5%)
IV-V,	0 (0.0%)
V,	1 (6.3%)
including Va,	1 (6.3%)
Vc,	0 (0.0%)
VI,	8 (50.0%)
including VIa,	1 (6.3%)
VIb,	5 (31.3%)
VIc	2 (12.5%)

Table SM3. Clinical characteristics of the MethylationEPIC and miRNA-seq cohort

Parameter	Patients with advanced carotid atherosclerosis (n=16)
**Clinical parameters**	
Men: Women	10:6
Age, years (M [Q1;Q3])	65.0 [62.5; 69.0]
BMI, kg/m^2 (M [Q1; Q3])	28.4 [26.6; 31.1]
Metabolic syndrome [n (%)]	15 (93.8%)
Waist circumference, cm (M [Q1;Q3])	*no data*
Carotid artery restenosis	1 (6.3%)
History of angina [n (%)]	11 (68.8%)
Angina functional class [n (%)]<br>- I FC<br>- II FC<br>- III FC	 2 (12.5%)<br>9 (56.3%)<br>0 (0.0%)
History of myocardial infarction [n (%)]	3 (18.8%)
History of coronary artery disease [n (%)]	14 (87.5%)
Duration of coronary artery disease, years (M [Q1;Q3])	8.0 [5.8; 9.3]
History of chronic heart failure [n (%)]	15 (93.8%)
Chronic heart failure functional class [n (%)]<br>- I FC<br>- II FC<br>- III FC	 0 (0.0%)<br>13 (81.3%)<br>0 (0.0%)
History of TIA or stroke [n (%)]	5 (31.3%)
Hypertension [n (%)]	16 (100.0%)
Duration of hypertension, years (M [Q1;Q3])	16.0 [10.0; 23.3]
Hypercholesterolemia [n (%)]	16 (100.0%)
Smoking [n (%)]<br>including former smokers	12 (75.0%)<br>0 (0.0%)
Diabetes mellitus [n (%)]	4 (25.0%)
Chronic limb ischemia [n (%)]	3 (18.8%)
**Laboratory data (M [Q1; Q3])**	
Glucose, mmol/l	5.7 [5.3; 7.6]
Total cholesterol, mmol/l	*no data*
Triglycerides, mmol/l	*no data*
LDL, mmol/l	*no data*
HDL, mmol/l	*no data*
Atherogenic index	*no data*
**Medications before hospitalization [n (%)]**	
Aspirin	16 (100.0%)
Clopidogrel	2 (12.5%)
Warfarin	0 (0.0%)
Beta-blockers	15 (93.8%)
Angiotensin converting enzyme inhibitors	9 (56.3%)
Angiotensin II receptor blockers	5 (31.3%)
Calcium channel blockers (dihydropyridine)	11 (68.8%)
Calcium channel blockers (non-dihydropyridine)	0 (0.0%)
Digoxin	0 (0.0%)
Loop diuretics	0 (0.0%)

Thiazide diuretics	2 (12.5%)
Spironolactone	1 (6.3%)
Nitrates	0 (0.0%)
Statins	16 (100.0%)
Cordarone	0 (0.0%)
Insulin	0 (0.0%)
Metformin	2 (12.5%)

Abbreviations: n, number of individuals in the group; M, median; Q1, first quartile (25th percentile); Q3, third quartile (75th percentile).

FC, functional class; HDL, high density lipoproteins; LDL, low density lipoproteins; TIA, transient ischemic attack.

3 Selection of miRNA and *MIR* gene regions for DNA methylation analysis.

We reviewed the publications indexed in HMDD v3.2: the Human miRNA Disease Database (http://www.cuilab.cn/hmdd) (Cui et al., 2024) and NCBI: National Center for Biotechnology Information (https://www.ncbi.nlm.nih.gov) and identified the microRNAs whose expression in the carotid artery tissue is associated with atherosclerotic lesion formation or plaque instability in at least two studies (**Table SM4**).

Table SM4. Expression of microRNAs associated with carotid atherosclerotic plaque instability in humans

№	miR	Unstable vs Stable Atherosclerotic Plaque	Study	Atherosclerotic plaque vs intact tissues	Study
**1**	-21	↓ S (TIA or stroke ≤6 weeks before CEA)	Markus B. et al., 2016	↑	Raitoharju E. et al., 2011; Cao J. et al., 2014; Markus B. et al., 2016
		↓ S (TIA or stroke ≤6 weeks before CEA), MRA (irregularities of plaque surface), H (ruptured)	Wang H. et al., 2019		
		↓ S (stroke or TIA within the past >14 days), H (ruptured)	Jin H. et al., 2018		
**2**	-100	↑ S	Cipollone F. et al., 2011; Maitrias P. et al., 2015	ND	
**3**	-127	↑ S	Cipollone F. et al., 2011; Maitrias P. et al., 2015	ND	
**4**	-133a	↑ S	Cipollone F. et al., 2011; Maitrias P. et al., 2015	ND	
**5**	-133b	↑ S	Cipollone F. et al., 2011	ND	
		↑ H (low-calcified)	Katano H. et al., 2018		
**6**	-143	↓ S (TIA or stroke ≤6 weeks before CEA)	Markus B. et al., 2016	↑	Markus B. et al., 2016
		↓ S (TIA or stroke ≤6 weeks before CEA), MRA (irregularities of plaque surface), H (ruptured)	Wang H. et al., 2019	↓	González-López et al., 2022
**7**	-145	↑ S	Cipollone F. et al., 2011; Maitrias P. et al., 2015	↓	Lovren F. et al., 2012
**8**	-146a	ND		↑	Raitoharju E. et al., 2011; Cao J. et al., 2014
9	-155	ND		↑	Raitoharju E. et al., 2011; Nazari-Jahantigh M. et al., 2012

Note. Atherosclerotic plaque instability was defined by symptoms (S), histology (H), or angiography (A). CEA, carotid endarterectomy; MRA, magnetic resonance angiography; ND, no data available; TIA, transient ischemic attack.

In addition, our previous work identified lower methylation of the CpG island located in the *HOXD4* promoter region, overlapping with the *MIR10B* gene body in the cells of coronary and carotid atherosclerotic plaques, and in peripheral blood leukocytes from patients with atherosclerosis, compared with unaffected internal thoracic arteries and great saphenous veins (Nazarenko et al., 2015). Accordingly, the genes encoding the nine miRNAs summarized in **Table SM5** (included miRNA host genes, such as *MIR143HG* (*CARMN*) for miR-143/145 cluster), together with *MIR10B*, were considered for further analysis of the methylation level within *MIR* genes regulatory regions.

Unfortunately, primer design and PCR optimization were unsuccessful for several candidate genes. Therefore, this ***Supplementary Matherial*** describe only the target regions and primers for which all experimental stages were completed and methylation data were obtained (**Table SM6**).

Table SM5. Candidate genes and regions for the analysis of miRNA regulatory elements

**miRNA**	**Gene (locus)**		**Coordinates* of the miRNA gene/ CpG island in the miRNA gene (number of CpG sites, if available)**	**Promoter ID and coordinates* according to FANTOM5**	**Coordinates* of the region selected for the methylation level analysis**		**miRNA location / host gene/ microRNA cluster**
miR-10b	*MIR10B*	2q31.1	chr2:177,015,031-177,015,140 / chr2:177,014,949-177,015,214 (18 CpG sites)	p1@HOXD3 chr2:177,001,291-177,001,315	*MIR10B*	chr2:177,014,629-177,015,244	Intergenic / *HOXD* cluster
miR-21	*MIR21*	17q23.1	chr17:57,918,627-57,918,698 / chr17:57,916,300-57,916,700 (16 CpG sites)	p1@AK310806 chr17:57,915,282-57,915,332	*MIR21* putative promoter	chr17:57,915,138-57,916,025	Intragenic / *VMP1* intron
					MIR21 CpG island	chr17:57,915,960-57,916,717	
					MIR21 gene body	chr17:57,918,142-57,918,840	
miR-100	*MIR100*	11q24.1	chr11:122,022,937-122,023,016	p2@ENST00000533109 chr11:122,051,328-122,051,348	chr11:122,024,891-122,025,506		Intragenic / *MIR100HG* intron
miR-127	*MIR127*	14q32.2	chr14:101,349,316-101,349,412 / chr14:101,349,373-101,349,860 (36 CpG sites)	p1@MEG3 chr14:101,292,449-101,292,462	chr14:101,349,259-101,349,884		Intergenic / *RTL1* imprinted region / miR-433/127 cluster
miR-143	*MIR143*	5q32	chr5:148,808,545-148,808,784	p1@MIR143HG chr5:148,786,424-148,786,453	chr5:148,786,304-148,786,864		Intragenic / noncoding RNA *CARMN* (*MIR143HG*) / miR-143/145 cluster
					chr5:148,808,001-148,808,583		
miR-145	*MIR145*		chr5:148,810,209-148,810,296		–		

* All coordinates are given according to the GRCh37/hg19 genome assembly

Table SM6: Studied regions and primer structure

**Region**	**Location relative to the gene**	**CpG-region category***	**Primer sequence**	**Primer annealing temperature**	**Number of CpG sites**	**Amplicon location** (according to GRCh37/hg19 assembly)	**PCR product length (bp)**
MIR10B	around the gene body	shore + CpG island	F: GTAGTAGGAAAAGGAGTAGATATAGGATATT<br>R: CCTCCTAAAATCCAAAATAATAAAACAAAA	62	23	chr2:177014629-177015244	616
			F: ATTTTGGTAGAAGAATGAGGGAATT<br>R: TTCTTTTCAACACCCAAAAAATACT	62	20	chr2:177014868-177015332	465
MIR21 putative promoter	upstream	open sea	F: TTTTTTGAGAAGAGGGGATAAGT<br>R: TCAAAACAACAACTCATTCTATATAACAAA	58	9	chr17:57915138-57916025	888
MIR21 CpG island	upstream	CpG island **	F: TTAAAAATAAGGGTAGAGTTAATGGAA<br>R: TTAATCAACTAAAAACAACAAAAATCC	60	17	chr17:57915960-57916717	758
MIR21 gene body	around the gene	open sea	F: GGGATTGGTTTATTTGGGGA<br>R: CCTCCATACTACTACATTATAACACAAAAA	62	6	chr17:57918142-57918840	699
MIR100 E-box	upstream	open sea	F: TTGATAAAGGTATGGGGTTAGAGGTA<br>R: AATTCTTACTTAATAAAACAAACCAAAAT	60	8	chr11: 122024891-122025506	616
MIR127	around the gene body	shore + CpG island	F: TTTTGGTATTTATAGGTTTTTAAGG<br>R: AATAAAAATAAACCCCTACCACAAC	60	40	chr14:101349259-101349884	626
MIR143HG promoter	upstream	open sea	F: GGGGTAGGTTAAGGTAAGGTAGTTA<br>R: AAACCAACAAAACCATAAAACTAAAAC	60	10	chr5:148786304-148786864	561
MIR143 gene body	5’ part of the gene	open sea	F: AGTATAAAGGTGGGGTATGGTATTAAA<br>R: ACAAAACAACTTCTCTCTTCCTAAACT	60	5	chr5:148808001-148808583	583

Note: *CpG-region categories were defined by CpG density. A **CpG island** is a dense cluster of CpG sites; a **shore** is a region up to 2 kb upstream or downstream from CpG island; and **open sea** is a region contained single CpG sites at a distance exceeding ± 4000 bp from the CpG island.** Classified as open sea according to UCSC browser.

According to the FANTOM5 database, the regulatory elements of the intergenic miRNAs *MIR10B* and *MIR127* are located at a distance from the miRNA gene bodies (~14 thousand bp upstream for *MIR10B* and ~57 thousand bp upstream for *MIR127*) (**Table SM5**). Previous studies have reported that atherosclerosis could alter the DNA methylation levels within regions in close proximity to the genes of these miRNAs [Aavik E. et al., 2014; Lacey M. et al., 2019b]. We therefore selected regions covering CpG islands that overlap the *MIR10B* and *MIR127* gene bodies. According to the UCSC Genome Browser (GRCh37/hg19 Assembly) (http://genome-euro.ucsc.edu), the CpG island in the *MIR10B* gene region is 266 bp at length (chr2:177014949-177015214) and contains 18 CpG sites, whereas the CpG island in the *MIR127* gene region is 488 bp at length (chr14:101349373-101349860) and contains 36 CpG sites.

For the intragenic miRNAs – *MIR21*, *MIR143* and *MIR145* – the reported locations of regulatory regions differed among publications and databases. We therefore visualized the reported coordinates in the UCSC Genome Browser and selected genomic regions that encompassed as many candidate regulatory elements as possible.

Most studies place the *MIR21* promoter within chr17:57914850-57915900 (Figure S1), whereas the *MIR21* gene body is located at chr17:57918627-57918698. Iorio M.V. et al. (2007) outlined a CpG island 2.5 kb upstream of the *MIR21* gene body, within approximately chr17:57916300-57916700. This 310-bp region met the following CpG island criteria: CpG content >50%, an observed-to-expected CpG ratio >0.6, and length >200 bp (Iorio et al., 2007). Nevertheless, applying the exact same criteria, the UCSC Genome Browser does not detect the CpG island 2.5 kb upstream the *MIR21* gene body. MethPrimer (https://www.urogene.org/cgi-bin/methprimer/methprimer.cgi) identified a 132-bp CpG island at chr17:57916335-57916466, with CpG content of 40% and an observed-to-expected CpG ratio >0.6. On this basis, we selected three regions for methylation analysis of the *MIR21* regulatory elements: the putative promoter of the *MIR21* gene, the CpG island and the *MIR21* gene body (**Table SM6**).

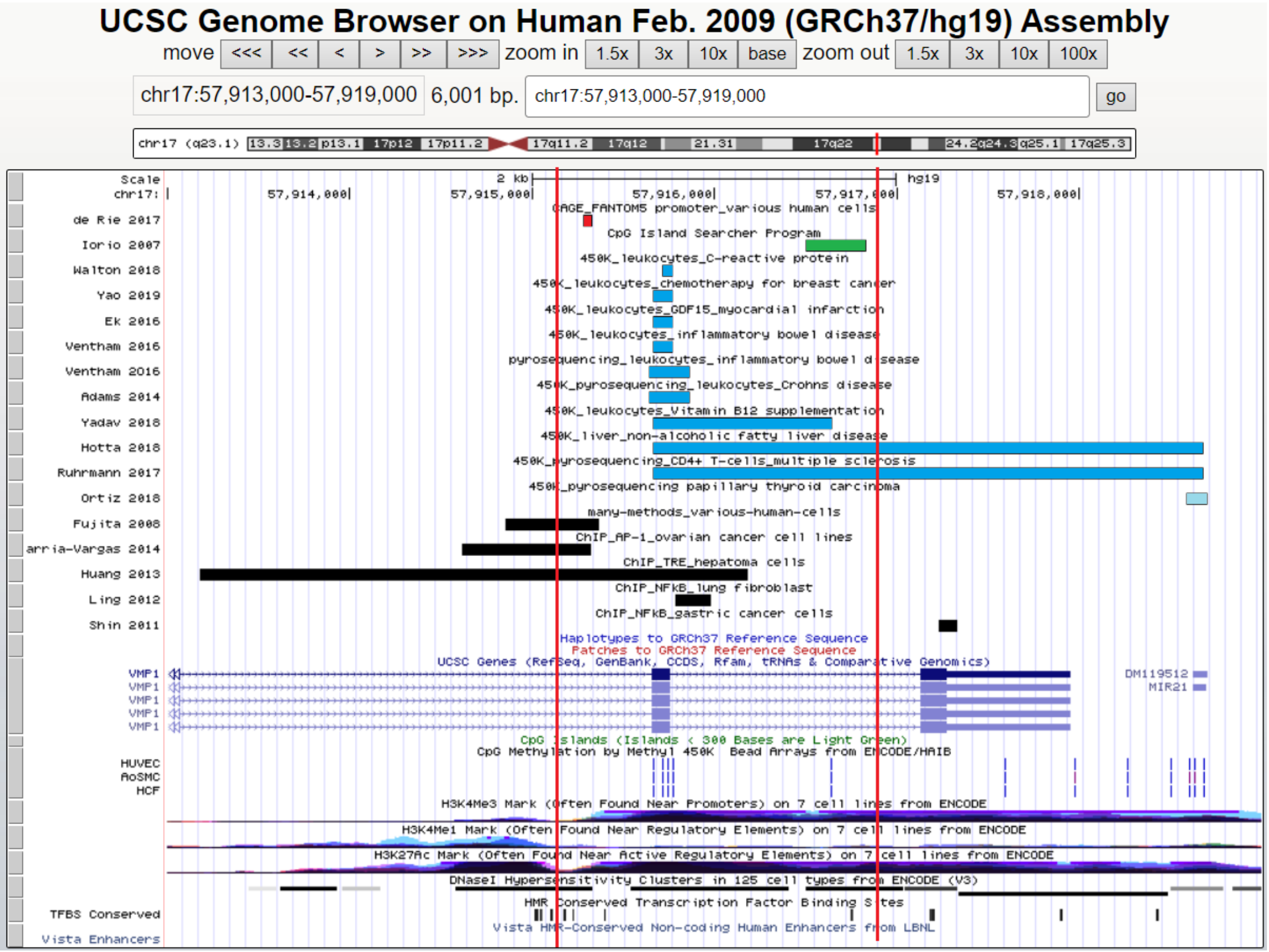

Supplementary Figure 1. Determining the location of regulatory elements of the *MIR21* gene.

The red box indicates the promoter region specified in the FANTOM5 database (http://fantom.gsc.riken.jp/5/) (de Rie et al., 2017), green box indicates the CpG island (Iorio et al., 2007), blue boxes indicate regions previously examined for DNA methylation by microarray or pyrosequencing in blood leukocytes and liver tissue in various diseases (Adams et al., 2014; Ek et al., 2016; Ventham et al., 2016; Hotta et al., 2018; Ruhrmann et al., 2018; Walton et al., 2018; Yadav et al., 2018; Yao et al., 2019), light blue box indicates the region demethylated after 5-azacytidine treatment of papillary thyroid carcinoma cells (Ortiz et al., 2018), black boxes indicate the putative promoters based on the computational approach (Fujita et al., 2008) and the chromatin-immunoprecipitation sequencing (ChIP-seq) data from the transcription factors search (Shin et al., 2010; Ling et al., 2012; Huang et al., 2013; Echevarría-Vargas et al., 2014). The vertical lines delimit the region selected for analysis.

Information about the own promoter of the *MIR143* gene is limited. The *MIR143* gene body is located at chr5:148808545-148808784. A 26-kb aorta/ovary/heart super-enhancer spanning the *CARMN* (*MIR143HG*) region has been reported to upregulate *MIR143* and *MIR145* irrespective of the precise transcription start sites of these miRNA genes and may be susceptible to disease-associated downregulation through DNA hypermethylation (Ehrlich et al., 2019. Other studies indicate that miR-143 expression may be regulated by the *MIR143HG* (miR-143 host gene) promoter at chr5:148786424-148786453 (**Figure S2**) (Blick et al., 2015; Deng et al., 2015; de Rie et al., 2017; Riches et al., 2017). We therefore selected the *MIR143HG* (*CARMN*) promoter region at chr5:148786304-148786864 (561 bp, 10 CpG sites) and the *MIR143* gene body region at chr5:148808001-148808583 (583 bp, 5 CpG sites) for methylation analysis.

For *MIR145*, the region selected to encompass as many putative promoter elements as possible (chr5:148810000-148810500) also overlapped the *MIR145* gene body (chr5:148810209-148810296) (**Figure S2**). Unfortunately, the of the *MIR145* gene body region (chr5:148809848-148810488) was unsuccessful.

No promoter location had been reported for the intragenic miRNA gene *MIR100*. Chen et al. (2014) showed that transcription factor *ZEB1* binds an E-box element that enhances miR-100 expression. This six-nucleotide motif (CAGCTG) is located 2.2 kb upstream of *MIR100* (Chen et al., 2014). According to the UCSC Genome Browser, the *MIR100* gene body spans chr11:122022937-122023016, and the E-box is located at chr11:122025183-122025188. We therefore selected chr11:122024891-122025506 (616 bp, 8 CpG sites), which fully encompasses the E-box, for methylation analysis. We also selected chr11:122022733-122023292 (560 bp, 7 CpG sites), which overlaps the *MIR100* gene body, but amplification of this region was not effective to obtain a product suitable for sequencing.

In total, eight regions were selected for DNA methylation analysis by BSAS (**Table SM6**): the CpG island overlapping the *MIR10B* gene body; the putative promoter, CpG island, and gene body of *MIR21*; the *MIR100* E-box region containing the *ZEB1*-binding site; the CpG island overlapping the *MIR127* gene body; the *MIR143HG* promoter; and the *MIR143* gene body.

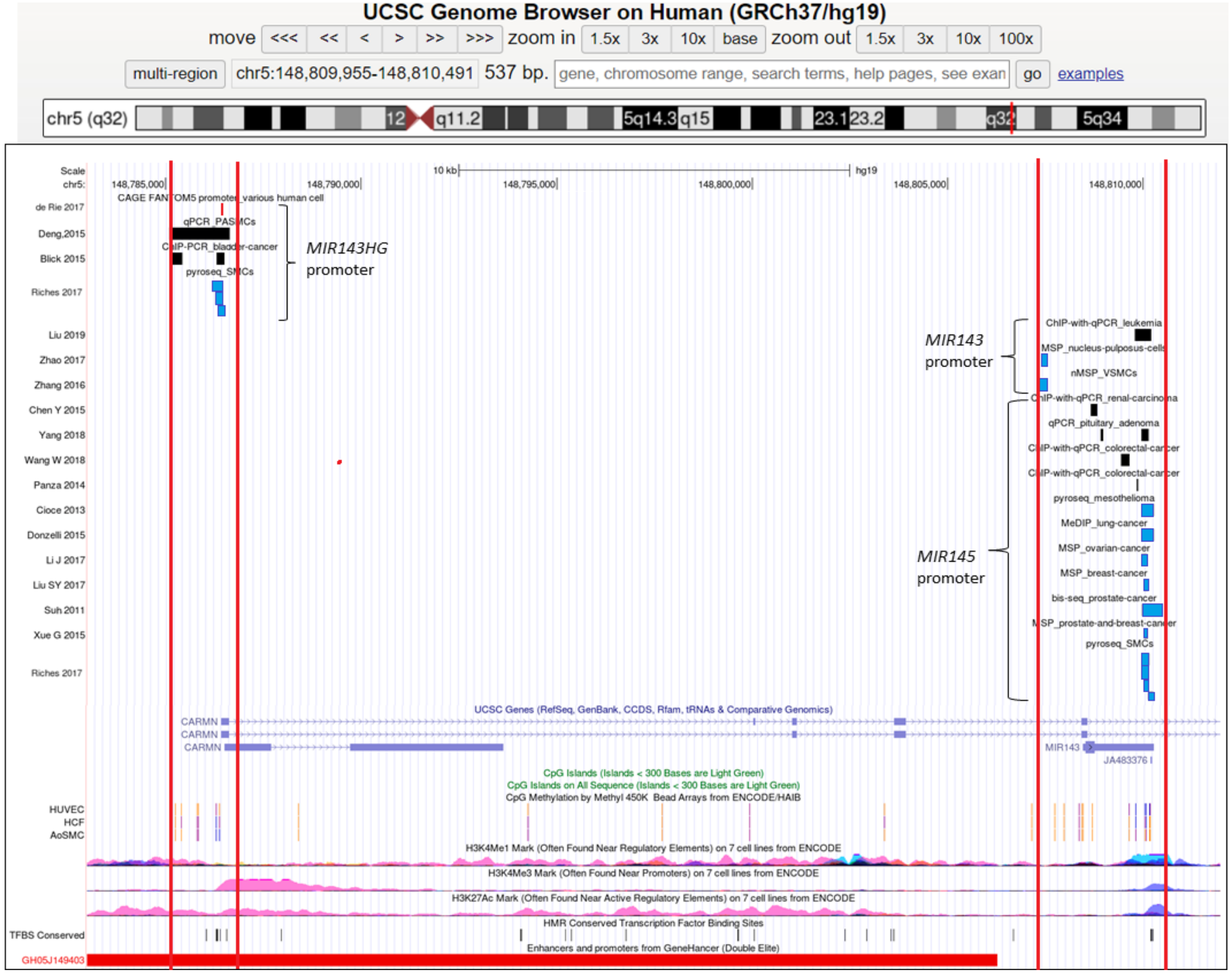

Supplementary Figure 2. Determining the location of regulatory elements of the *MIR143* and *MIR145* genes.

The red box indicates the promoter region specified in the FANTOM5 database (http://fantom.gsc.riken.jp/5 /) (de Rie D. et al., 2017), blue boxes indicate the regions previously examined for DNA methylation in smooth muscle cells (Zhang et al., 2016; Riches et al., 2017), nucleus pulposus cells (Zhao et al., 2017) and in various cancer cells (Suh et al., 2011; Cioce et al., 2013; Donzelli et al., 2015; Xue et al., 2015; Li et al., 2017; Liu J. et al., 2017; Liu S.Y. et al., 2017), black boxes indicate the regions proposed as *MIR143* or *MIR145* promoters, including findings based on the chromatin-immunoprecipitation sequencing (ChIP-seq) data from the transcription factors search (Panza et al., 2014; Blick et al., 2015; Chen et al., 2015; Deng et al., 2015; Wang et al., 2018; Yang et al., 2018; Liu et al., 2019). The vertical lines delimit the region selected for analysis.

4 DNA methylation analysis by Bisulfite Amplicon Sequencing (BSAS)

DNA methylation analysis by high-throughput MPS included the following steps: (1) selection of target genomic regions and primer design; (2) amplification of target regions by PCR; (3) equimolar pooling and purification of amplicons; (4) DNA library preparation and sequencing; (5) bioinformatics processing of the results.

4.1 Primer design for BSAS. Primers were designed several publicly available bioinformatic tools. MethPrimer (https://www.methprimer.com/) was used with the following criteria: primer length, 20-30 nucleotides; melting temperature, 58-65°C; and product size, 300-900 nucleotides (Li, Dahiya, 2002). The theoretical melting temperature and GC content of the oligonucleotide sequence, as well as the possibility of hairpins, self- and hetero-dimers of primers were evaluated using the IDT OligoAnalyzer online tool (https://eu.idtdna.com/calc/analyzer). Amplicon specificity for the target region was determined by *in silico* PCR using BiSearch ePCR online tool (http://bisearch.enzim.hu/?m=genompsearch). Primer sequences are provided in in **Table SM6**.

4.2 Amplification of target DNA regions. Target regions were amplified using the BioMaster HS-Taq PCR-Color (2×) PCR kit (BioLabMix, Russia). The optimal annealing temperature was determined by gradient PCR, varying the temperature within ±2°C of the calculated melting temperature for each primer pair. DNA amplification was performed on ProFlex PCR System (ThermoFisher Scientific, USA) and DTPrime4 (DNA-Technology, Russia) using the following program: 95°C for 5 min, 40 cycles of 95°C for 30 s, primer-specific annealing temperature for 30 s, and 72°C for 60 s; followed by 72°C for 5 min. Annealing temperatures for individual primer pairs are listed in **Table SM6**. The PCR products were evaluated by DNA electrophoresis in 3% agarose gel stained with ethidium bromide and visualized using a ChemiDoc XRS+ System or Gel Doc XR+ Imaging System (Bio-Rad, USA).

4.3 Equimolar pooling and purification of amplicons. Amplicons from each DNA sample were pooled equimolarly (approximately 200 billion copies of double-stranded DNA per amplicon) and diluted in 200 uL. Pooled amplicons were purified for library preparation using the GeneJET NGS PCR Product Cleanup Kit (ThermoFisher Scientific, USA). The concentration of purified amplicons was measured on a Qubit 3.0 fluorimeter (ThermoFisher Scientific, USA).

4.4 DNA library preparation and sequencing. After DNA concentration measurement, purified amplicon pools were used to prepare DNA libraries with the Nextera XT DNA Library Prep Kit or Nextera DNA Flex Library Prep Kit (Illumina, USA) according to the manufacturer's protocol. Pooled libraries were sequenced on a MiSeq (Illumina, USA) using MiSeq Reagent Kit v2 (300-cycles, Illumina, USA) in the sequencing mode of 150-bp paired-end reads.

4.5 Bioinformatic processing of the results. The *methylseq* pipeline implemented in Nextflow (Ewels P.A. et al., 2020) was used to automate and standardize data processing. The resulting reads of DNA fragments were automatically sorted into samples with the nucleotide sequence of the adapters removed. Reads were filtered for quality (minimum Q30) and length (minimum 20 nucleotides) using the *Trim Galore* tool (Krueger et al., 2021), then aligned to the GRCh37/hg19 human reference genome using the *bwa-meth* tool (Pedersen et al., 2014). CpG methylation information was extracted from BAM files using Bismark tool (Krueger, Andrews, 2011). FastQC

(https://www.bioinformatics.babraham.ac.uk/projects/fastqc/), Qualimap, Preseq, and MultiQC tools were used for quality control (Daley, Smith, 2013; Ewels et al., 2016; Okonechnikov et al., 2016).

Subsequent processing of locus-specific methylation profiles and statistical analysis were performed in R using methylKit (Akalin A. et al., 2012). CpG-site methylation was defined as the percentage of reads containing methylated cytosine relative to all cytosine reads at the corresponding genomic position. CpG sites with less than 15-fold coverage were excluded from the analysis.

5 Methods for microRNA expression analysis

5.1 RNA extraction. Total RNA, including microRNAs, was extracted from fragments of the same 16 carotid artery samples previously utilized for the microarray-based DNA methylation analysis (Table SM3). Tissues were preliminarily homogenized using a Minilys homogenizer (Bertin Technologies, France) in TRIzol reagent (Thermo Fisher Scientific, USA), followed by isolation according to the manufacturer's protocol. The quality of the extracted RNA was assessed using an Agilent 2100 Bioanalyzer (Agilent Technologies, USA). Following quality assessment, the RNA samples were frozen and stored at −80°C until further analysis.

5.2 MicroRNA sequencing (miRNA-seq). MicroRNA sequencing libraries were prepared using the NEBNext Multiplex Small RNA Library Prep Set for Illumina (New England Biolabs, USA), following the manufacturer's protocol. Library preparation included 3′ SR adapter ligation, hybridization of the reverse transcription primer, 5′ SR adapter ligation, reverse transcription, PCR amplification, and size selection of small RNAs. Amplification using indexed NEBNext SR primers was performed on a ProFlex PCR System (Thermo Fisher Scientific, USA) using the following cycling conditions: 94°C for 30 s; 12 cycles of 94°C for 15 s, 62°C for 15 s, and 70°C for 15 s; followed by a final extension at 70°C for 5 min. The amplified libraries were purified using the Monarch PCR & DNA Cleanup Kit (New England Biolabs, USA).

Small RNA fragments were size-selected using AMPure XP magnetic beads (Beckman Coulter, USA). The quality of the prepared libraries was assessed using an Agilent 2100 Bioanalyzer (Agilent Technologies, USA). The resulting cDNA libraries were sequenced on a HiSeq 1500 platform (Illumina, USA) in single-end mode with a read length of 50 nucleotides.

5.3 Bioinformatic processing of the results.

Sequencing data were processed and microRNA expression was quantified using the miARma-Seq software package (Andrés-León E. et al., 2018). Read quality was assessed using FastQC v0.11.5. Adapter sequences were removed and reads were trimmed using Trim Galore (Krueger F. et al., 2021). The resulting sequences were aligned to the human reference genome assembly GRCh37/hg19 using BWA-MEM (Li H., 2013). The number of reads assigned to microRNAs was determined using the featureCounts algorithm (Liao Y. et al., 2014). MicroRNA expression levels were quantified using the edgeR package in the R statistical computing environment and expressed as counts per million (CPM) mapped reads for each sample. MicroRNAs with an expression level of at least 10 CPM in at least two samples were retained for downstream analysis.

References

1. Aavik, E., Lumivuori, H., Leppänen, O., Wirth, T., Häkkinen, S. K., Bräsen, J.H., et al. (2015) Global DNA methylation analysis of human atherosclerotic plaques reveals extensive genomic hypomethylation and reactivation at imprinted locus 14q32 involving induction of a microRNA cluster. *Eur. Heart J.* 36 (16), 993–1000. doi:10.1093/eurheartj/ehu437
2. Adams, A. T., Kennedy, N. A., Hansen, R., Ventham, N. T., O'Leary, K. R., Drummond, H. E., et al. (2014). Two-stage Genome-wide Methylation Profiling in Childhood-onset Crohn's Disease Implicates Epigenetic Alterations at the VMP1/MIR21 and HLA Loci: *Inflammatory Bowel Diseases* 20, 1784–1793. doi:10.1097/MIB.0000000000000179
3. Akalin, A., Kormaksson, M., Li, S., Garrett-Bakelman, F. E., Figueroa, M. E., Melnick, A., et al. (2012). methylKit: a comprehensive R package for the analysis of genome-wide DNA methylation profiles. *Genome Biol* 13, R87. doi:10.1186/gb-2012-13-10-r87
4. Andrés-León, E., Núñez-Torres, R., and Rojas, A. M. (2016). miARma-Seq: a comprehensive tool for miRNA, mRNA and circRNA analysis. Sci Rep 6, 25749. doi:10.1038/srep25749
5. Blick, C., Ramachandran, A., McCormick, R., Wigfield, S., Cranston, D., Catto, J., et al. (2015). Identification of a hypoxia-regulated miRNA signature in bladder cancer and a role for miR-145 in hypoxia-dependent apoptosis. *Br J Cancer* 113, 634–644. doi:10.1038/bjc.2015.203
6. Cao, J., Zhang, K., Zheng, J., and Dong, R. (2015). MicroRNA-146a and -21 cooperate to regulate vascular smooth muscle cell proliferation via modulation of the Notch signaling pathway. *Mol. Med. Rep*. 11 (4), 2889–2895. doi:10.3892/mmr.2014.3107
7. Chen, D., Sun, Y., Yuan, Y., Han, Z., Zhang, P., Zhang, J., et al. (2014). miR-100 Induces Epithelial-Mesenchymal Transition but Suppresses Tumorigenesis, Migration and Invasion. *PLoS Genet* 10, e1004177. doi:10.1371/journal.pgen.1004177
8. Chen, Y., Sun, Y., Rao, Q., Xu, H., Li, L., and Chang, C. (2015). Androgen receptor (AR) suppresses miRNA-145 to promote renal cell carcinoma (RCC) progression independent of VHL status. *Oncotarget* 6, 31203–31215. doi:10.18632/oncotarget.4522
9. Cioce, M., Ganci, F., Canu, V., Sacconi, A., Mori, F., Canino, C., et al. (2014). Protumorigenic effects of mir-145 loss in malignant pleural mesothelioma. *Oncogene* 33, 5319–5331. doi:10.1038/onc.2013.476
10. Cipollone, F., Felicioni, L., Sarzani, R., Ucchino, S., Spigonardo, F., Mandolini, C., et al. (2011). A Unique MicroRNA Signature Associated With Plaque Instability in Humans. *Stroke* 42 (9), 2556–2563. doi:10.1161/STROKEAHA.110.597575
11. Cui, C., Zhong, B., Fan, R., and Cui, Q. (2024). HMDD v4.0: a database for experimentally supported human microRNA-disease associations. *Nucleic Acids Res.* 52 (D1), D1327–D1332. doi:10.1093/nar/gkad717
12. Daley, T., and Smith, A. D. (2013). Predicting the molecular complexity of sequencing libraries. *Nat Methods* 10, 325–327. doi:10.1038/nmeth.2375
13. De Rie, D., Abugessaisa, I., Alam, T., Arner, E., Arner, P., et al. (2017). An integrated expression atlas of miRNAs and their promoters in human and mouse. *Nat. Biotechnol.* 35 (9), 872–878. doi:10.1038/nbt.3947
14. Deng, L., Blanco, F. J., Stevens, H., Lu, R., Caudrillier, A., McBride, M., et al. (2015). MicroRNA-143 Activation Regulates Smooth Muscle and Endothelial Cell Crosstalk in Pulmonary Arterial Hypertension. *Circulation Research* 117, 870–883. doi:10.1161/CIRCRESAHA.115.306806
15. Donzelli, S., Mori, F., Bellissimo, T., Sacconi, A., Casini, B., Frixa, T., et al. (2015). Epigenetic silencing of miR-145-5p contributes to brain metastasis. *Oncotarget* 6, 35183–35201. doi:10.18632/oncotarget.5930

16. Echevarría-Vargas, I. M., Valiyeva, F., and Vivas-Mejía, P. E. (2014). Upregulation of miR-21 in Cisplatin Resistant Ovarian Cancer via JNK-1/c-Jun Pathway. *PLoS ONE* 9, e97094. doi:10.1371/journal.pone.0097094
17. Ehrlich, K. C., Lacey, M., and Ehrlich, M. (2019). Tissue-Specific Epigenetics of Atherosclerosis-Related *ANGPT* and *ANGPTL* Genes. *Epigenomics* 11 (2), 169–186. doi:10.2217/epi-2018-0150
18. Ek, W. E., Hedman, Å. K., Enroth, S., Morris, A. P., Lindgren, C. M., Mahajan, A., et al. (2016). Genome-wide DNA methylation study identifies genes associated with the cardiovascular biomarker GDF-15. *Hum. Mol. Genet.* 25 (4), 817–827. doi:10.1093/hmg/ddv511
19. Ewels, P., Magnusson, M., Lundin, S., and Käller, M. (2016). MultiQC: summarize analysis results for multiple tools and samples in a single report. *Bioinformatics* 32, 3047–3048. doi:10.1093/bioinformatics/btw354
20. Fujita, S., Ito, T., Mizutani, T., Minoguchi, S., Yamamichi, N., Sakurai, K., et al. (2008). miR-21 Gene Expression Triggered by AP-1 Is Sustained through a Double-Negative Feedback Mechanism. *Journal of Molecular Biology* 378, 492–504. doi:10.1016/j.jmb.2008.03.015
21. Gao, Y., Feng, C., Zhang, Y., Song, C., Chen, J., Li, Y., et al. (2022). TRmir: A Comprehensive Resource for Human Transcriptional Regulatory Information of MicroRNAs. *Front Genet*. 13, 808950. doi:10.3389/fgene.2022.808950
22. González-López, P., Ares-Carral, C., López-Pastor, A. R., Infante-Menéndez, J., González Illaness, T., Vega De Ceniga, M., et al. (2022). Implication of miR-155-5p and miR-143-3p in the Vascular Insulin Resistance and Instability of Human and Experimental Atherosclerotic Plaque. *IJMS*. 23 (18), 10253. doi:10.3390/ijms231810253
23. Hotta, K., Kitamoto, T., Kitamoto, A., Ogawa, Y., Honda, Y., Kessoku, T., et al. (2018). Identification of the genomic region under epigenetic regulation during non-alcoholic fatty liver disease progression. *Hepatology Research* 48. doi:10.1111/hepr.12992
24. Huang, Y.-H., Lin, Y.-H., Chi, H.-C., Liao, C.-H., Liao, C.-J., Wu, S.-M., et al. (2013). Thyroid Hormone Regulation of miR-21 Enhances Migration and Invasion of Hepatoma. *Cancer Research* 73, 2505–2517. doi:10.1158/0008-5472.CAN-12-2218
25. Iorio, M. V., Visone, R., Di Leva, G., Donati, V., Petrocca, F., Casalini, P., et al. (2007). MicroRNA Signatures in Human Ovarian Cancer. *Cancer Res.* 67 (18), 8699–8707. doi:10.1158/0008-5472.CAN-07-1936.
26. Jin, H., Li, D. Y., Chernogubova, E., Sun, C., Busch, A., Eken, S. M., et al. (2018). Local Delivery of miR-21 Stabilizes Fibrous Caps in Vulnerable Atherosclerotic Lesions. *Molecular Therapy* 26, 1040–1055. doi:10.1016/j.ymthe.2018.01.011
27. Katano, H., Nishikawa, Y., Yamada, H., Yamada, K., and Mase, M. (2018). Differential Expression of microRNAs in Severely Calcified Carotid Plaques. *J. Stroke Cerebrovasc. Dis.* 27 (1), 108–117. doi:10.1016/j.jstrokecerebrovasdis.2017.08.009
28. Krueger F., Andrews S. R. (2011) Bismark: a flexible aligner and methylation caller for Bisulfite-Seq applications. *Bioinformatics*. 27(11):1571–1572.
29. Krueger F. et al. FelixKrueger/TrimGalore: v0.6.7. – 2021 doi:10.5281/zenodo.5127898
30. Lacey, M., Baribault, C., Ehrlich, K. C., and Ehrlich, M. (2019). Data showing atherosclerosis-associated differentially methylated regions are often at enhancers. *Data in Brief* 23, 103812. doi:10.1016/j.dib.2019.103812
31. Li, H. (2013) Aligning sequence reads, clone sequences and assembly contigs with BWA-MEM. *ArXiv* 1303.

32. Li, J., Lu, J., Ye, Z., Han, X., Zheng, X., Hou, H., et al. (2017). 20(S)-Rg3 blocked epithelial-mesenchymal transition through DNMT3A/miR-145/FSCN1 in ovarian cancer. *Oncotarget* 8, 53375–53386. doi:10.18632/oncotarget.18482
33. Li, L.-C., and Dahiya, R. (2002). MethPrimer: designing primers for methylation PCRs. *Bioinformatics* 18, 1427–1431. doi:10.1093/bioinformatics/18.11.1427
34. Liao, Y., Smyth, G. K., and Shi, W. (2014). featureCounts: an efficient general purpose program for assigning sequence reads to genomic features. Bioinformatics 30, 923–930. doi:10.1093/bioinformatics/btt656
35. Ling, M., Li, Y., Xu, Y., Pang, Y., Shen, L., Jiang, R., et al. (2012). Regulation of miRNA-21 by reactive oxygen species-activated ERK/NF-κB in arsenite-induced cell transformation. *Free Radical Biology and Medicine* 52, 1508–1518. doi:10.1016/j.freeradbiomed.2012.02.020
36. Liu, J., Li, M., Wang, Y., and Luo, J. (2017). Curcumin sensitizes prostate cancer cells to radiation partly via epigenetic activation of miR-143 and miR-143 mediated autophagy inhibition. *Journal of Drug Targeting* 25, 645–652. doi:10.1080/1061186X.2017.1315686
37. Liu, S.-Y., Li, X.-Y., Chen, W.-Q., Hu, H., Luo, B., Shi, Y.-X., et al. (2017). Demethylation of the *MIR145* promoter suppresses migration and invasion in breast cancer. *Oncotarget* 8, 61731–61741. doi:10.18632/oncotarget.18686
38. Liu, T., Xia, L., Yao, Y., Yan, C., Fan, Y., Gajendran, B., et al. (2019). Identification of diterpenoid compounds that interfere with Fli-1 DNA binding to suppress leukemogenesis. *Cell Death Dis* 10, 117. doi:10.1038/s41419-019-1363-1
39. Lovren, F., Pan, Y., Quan, A., Singh, K. K., Shukla, P. C., Gupta, N., et al. (2012). MicroRNA-145 Targeted Therapy Reduces Atherosclerosis. *Circulation* 126 (11, Suppl. 1), S81-S90. doi:10.1161/CIRCULATIONAHA.111.084186
40. Maitrias, P., Metzinger-Le Meuth, V., Massy, Z. A., M'Baya-Moutoula, E., Reix, T., Caus, T., et al. (2015). MicroRNA deregulation in symptomatic carotid plaque. *J. Vasc. Surg.* 62 (5),1245-1250.e1. doi:10.1016/j.jvs.2015.06.136
41. Markus, B., Grote, K., Worsch, M., Parviz, B., Boening, A., Schieffer, B., et al. (2016). Differential Expression of MicroRNAs in Endarterectomy Specimens Taken from Patients with Asymptomatic and Symptomatic Carotid Plaques. *PLoS ONE* 11 (9), e0161632. doi:10.1371/journal.pone.0161632
42. Nazarenko, M. S., Markov, A. V., Lebedev, I. N., Freidin, M. B., Sleptcov, A. A., Koroleva, I. A., et al. (2015). A Comparison of Genome-Wide DNA Methylation Patterns between Different Vascular Tissues from Patients with Coronary Heart Disease. *PLoS ONE* 10 (4), e0122601. doi:10.1371/journal.pone.0122601
43. Nazari-Jahantigh, M., Wei, Y., Noels, H., Akhtar, S., Zhou, Z., Koenen, R. R., et al. (2012). MicroRNA-155 promotes atherosclerosis by repressing Bcl6 in macrophages. J. Clin. Invest. 122 (11), 4190–4202. doi:10.1172/JCI61716
44. Okonechnikov K., Conesa A., Garcia-Alcalde F. (2016) Qualimap 2: advanced multi-sample quality control for high-throughput sequencing data. *Bioinformatics*. 2(2):292–294.
45. Ortiz, I. M. D. P., Barros-Filho, M. C., Dos Reis, M. B., Beltrami, C. M., Marchi, F. A., Kuasne, H., et al. (2018). Loss of DNA methylation is related to increased expression of miR-21 and miR-146b in papillary thyroid carcinoma. *Clin Epigenet* 10, 144. doi:10.1186/s13148-018-0579-8
46. Panza, A., Votino, C., Gentile, A., Valvano, M. R., Colangelo, T., Pancione, M., et al. (2014). Peroxisome proliferator-activated receptor γ-mediated induction of microRNA-145 opposes tumor phenotype in colorectal cancer. Biochimica et Biophysica Acta (BBA) - Molecular Cell Research 1843, 1225–1236. doi:10.1016/j.bbamcr.2014.03.003

47. Pedersen, B. S., Eyring, K., De, S., Yang, I. V., and Schwartz, D. A. (2014). Fast and accurate alignment of long bisulfite-seq reads. *arXiv: Genomics*. doi:10.48550/ARXIV.1401.1129
48. Raitoharju, E., Lyytikäinen, L.-P., Levula, M., Oksala, N., Mennander, A., Tarkka, M., et al. (2011). miR-21, miR-210, miR-34a, and miR-146a/b are up-regulated in human atherosclerotic plaques in the Tampere Vascular Study. *Atherosclerosis* 219, 211–217. doi:10.1016/j.atherosclerosis.2011.07.020
49. Riches, K., Huntriss, J., Keeble, C., Wood, I. C., O'Regan, D. J., Turner, N. A., et al. (2017). Mapping the methylation status of the miR-145 promoter in saphenous vein smooth muscle cells from individuals with type 2 diabetes. *Diab. Vasc. Dis. Res.*14 (2), 122–129. doi:10.1177/1479164116677968
50. Ruhrmann, S., Ewing, E., Piket, E., Kular, L., Cetrulo Lorenzi, J. C., Fernandes, S. J., et al. (2018). Hypermethylation of *MIR21* in CD4+ T cells from patients with relapsing-remitting multiple sclerosis associates with lower miRNA-21 levels and concomitant up-regulation of its target genes. *Mult Scler* 24, 1288–1300. doi:10.1177/1352458517721356
51. Shin, V. Y., Jin, H., Ng, E. K. O., Cheng, A. S. L., Chong, W. W. S., Wong, C. Y. P., et al. (2011). NF-κB targets miR-16 and miR-21 in gastric cancer: involvement of prostaglandin E receptors. *Carcinogenesis* 32, 240–245. doi:10.1093/carcin/bgq240
52. Suh, S. O., Chen, Y., Zaman, M. S., Hirata, H., Yamamura, S., Shahryari, V., et al. (2011). MicroRNA-145 is regulated by DNA methylation and p53 gene mutation in prostate cancer. *CARCIN* 32, 772–778. doi:10.1093/carcin/bgr036
53. Ventham, N. T., Kennedy, N. A., Adams, A. T., Kalla, R., Heath, S., O'Leary, K. R., et al. (2016). Integrative epigenome-wide analysis demonstrates that DNA methylation may mediate genetic risk in inflammatory bowel disease. *Nat Commun* 7, 13507. doi:10.1038/ncomms13507
54. Walton, E., Hemani, G., Dehghan, A., Relton, C., and Smith, G. D. (2018). Systematic evaluation of the causal relationship between DNA methylation and C-reactive protein. *bioRxiv*. 397836. doi:10.1101/397836
55. Wang, H., Liu, D., and Zhang, H. (2019). Investigation of the Underlying Genes and Mechanism of Macrophage-Enriched Ruptured Atherosclerotic Plaques Using Bioinformatics Method. *JAT* 26, 636–658. doi:10.5551/jat.45963
56. Wang, W., Xiao, X., Chen, X., Huo, Y., Xi, W., Lin, Z., et al. (2018). Tumor-suppressive miR-145 co-repressed by TCF4-β-catenin and PRC2 complexes forms double-negative regulation loops with its negative regulators in colorectal cancer. *Intl Journal of Cancer* 142, 308–321. doi:10.1002/ijc.31056
57. Xue, G., Ren, Z., Chen, Y., Zhu, J., Du, Y., Pan, D., et al. (2015). A feedback regulation between miR-145 and DNA methyltransferase 3b in prostate cancer cell and their responses to irradiation. *Cancer Letters* 361, 121–127. doi:10.1016/j.canlet.2015.02.046.
58. Yadav, D. K., Shrestha, S., Lillycrop, K. A., Joglekar, C. V., Pan, H., Holbrook, J. D., et al. (2018). Vitamin B_{12} Supplementation Influences Methylation of Genes Associated with Type 2 Diabetes and Its Intermediate Traits. *Epigenomic* 10, 71–90. doi:10.2217/epi-2017-0102
59. Yang, W., Xu, T., Qiu, P., and Xu, G. (2018). Caveolin-1 promotes pituitary adenoma cells migration and invasion by regulating the interaction between EGR1 and KLF5. *Experimental Cell Research* 367, 7–14. doi:10.1016/j.yexcr.2018.01.008
60. Yao, S., Hu, Q., Kerns, S., Yan, L., Onitilo, A. A., Misleh, J., et al. (2019). Impact of chemotherapy for breast cancer on leukocyte DNA methylation landscape and cognitive function: a prospective study. *Clin Epigenet* 11, 45. doi:10.1186/s13148-019-0641-1
61. Zhang, H.-P., Wang, Y.-H., Cao, C.-J., Yang, X.-M., Ma, S.-C., Han, X.-B., et al. (2016). A regulatory circuit involving miR-143 and DNMT3a mediates vascular smooth muscle cell

proliferation induced by homocysteine. *Molecular Medicine Reports* 13, 483–490. doi:10.3892/mmr.2015.4558

62. Zhao, K., Zhang, Y., Kang, L., Song, Y., Wang, K., Li, S., et al. (2017). Epigenetic silencing of miRNA-143 regulates apoptosis by targeting BCL2 in human intervertebral disc degeneration. *Gene* 628, 259–266. doi:10.1016/j.gene.2017.07.043
